# Digital Primary Health in Rwanda: Qualitative Study of User Experiences and Implementation Lessons From Babyl’s Telemedicine Platform

**DOI:** 10.2196/84832

**Published:** 2026-04-01

**Authors:** Sabine Musange Furere, Gashaija Absolomon, Nathalie Umutoni, Stella Matutina Umuhoza, Felix K Rubuga, Yvonne Delphine Nsaba-Uwera, Patrick Singa Muhoza, Regis Hitimana, Jeanine Condo, James Humuza

**Affiliations:** 1 Centre for Impact, Innovation and Capacity Building for Health Information Systems and Nutrition (CIIC-HIN), Kigali, Rwanda; 2 School of Public Health, University of Rwanda, KK 19 Ave, Kigali, P.O. Box 4285, Rwanda, 250 788307858; 3 Partners In Health - Inshuti Mu Buzima, Kigali, Rwanda; 4 School of Nursing and Midwifery, University of Rwanda, Kigali, Rwanda; 5 Irembo, Kigali, Rwanda; 6 Rwanda Social Security Board (RSSB), Kigali, Rwanda; 7 Tulane School of Public Health and Tropical Medicine, New Orleans, LA, United States

**Keywords:** digital health, telemedicine, implementation science, qualitative research, Rwanda, low- and middle-income countries, health system integration

## Abstract

**Background:**

Digital health innovations address health care accessibility challenges in low- and middle-income countries. Babyl, Rwanda’s largest telemedicine platform, reached 450 of 510 health facilities and enrolled 2 million patients before halting in September 2023 for system redesign. Limited research has explored implementation experiences and user perspectives that influenced its sustainability.

**Objective:**

This study aims to explore user experiences and implementation lessons from Babyl’s digital health platform, examining drivers that supported or hindered adoption and scale-up. This qualitative study uniquely examines the lived experiences of diverse stakeholders, active users, lapsed users, nonusers, health care providers, and Babyl agents to understand implementation challenges that contributed to the platform’s halt.

**Methods:**

A qualitative, cross-sectional study used 20 focus group discussions (FGDs) and 32 key informant interviews (KIIs) across 12 health centers in ten districts with diverse utilization rates, geographic locations, and Babyl agent availability. FGDs captured collective community perspectives while KIIs provided in-depth individual experiences, enabling data triangulation. FGDs included active users, lapsed users, registered nonusers, and eligible nonregistrants. KIIs involved health center heads, health care providers, and Babyl agents. Data were analyzed using thematic analysis following Braun and Clarke’s framework. Data saturation was achieved when no new themes emerged from the last 3 FGDs and 5 KIIs. All transcripts were validated through member checking with a subset of participants, and intercoder reliability was established with a Cohen kappa of 0.82 across 2 independent coders.

**Results:**

Five themes emerged: (1) knowledge and perceptions of digital health, (2) enablers and barriers to utilization, (3) experience and satisfaction, (4) benefits, and (5) improvement suggestions. Participants held positive perceptions of digital health for improving access and reducing wait times. Key enablers included qualified providers, convenience, privacy, and Babyl agents. Major barriers included negative perceptions of remote care quality, service delays, limited digital literacy, device access challenges, and inadequate health facility integration. Users reported high satisfaction with consultations but experienced process confusion. Patient and provider perspectives diverged: patients emphasized convenience, while providers expressed concerns about diagnostic limitations without physical examination. Digital literacy and smartphone access were pronounced barriers among rural and older participants. Recommendations included community mobilization, universal agent deployment, expanded coverage, and sustainable financing.

**Conclusions:**

Multiple implementation challenges at individual, community, health system, and policy levels contributed to Babyl’s discontinuation. Critical lessons include the importance of genuine health system integration, sustainable financing, stakeholder engagement, and gradual scaling. Findings provide insights for Rwanda’s health sector digitalization and other African nations investing in telemedicine platforms.

## Introduction

Digital health innovations have emerged as transformative solutions to address health care accessibility challenges, particularly in low- and middle-income countries (LMICs) where geographic barriers, provider shortages, and infrastructure limitations impede effective health care delivery [1]. The World Health Assembly of the World Health Organization (WHO) unanimously adopted the use of telehealth in 2005 as a means of improving access to specialized health care services and contributing towards achieving Universal Health Coverage goals [2,3]. The expansion of mobile phone usage in emerging markets has created unprecedented opportunities for telemedicine platforms to revolutionize health care access and convenience, offering remote consultation services that can bridge the gap between patients and health care providers [4,5].

Rwanda’s health care landscape exemplifies both the potential and challenges of implementing digital health solutions at scale. The country’s well-structured health care system operates through a hierarchical model, with 499 health centers (HCs) serving as the backbone of primary health care delivery, addressing approximately 80% of the disease burden at the peripheral level [6,7]. Each HC provides a package of services, including curative, preventive, promotional, and rehabilitation services, while supervising health posts and community health workers in their catchment areas. This systematic approach to health care delivery created a conducive environment for digital health integration.

Babyl, an innovative digital health care platform, was introduced in Rwanda with the mission to transform health care service delivery through digital consultations facilitated by experienced health care providers. Leveraging Rwanda’s high mobile phone penetration rate, Babyl’s platform connected citizens with health care professionals through artificial intelligence–powered chatbots that triaged patients’ medical concerns, offered treatment recommendations, and facilitated appointments with remote physicians based on symptom severity [7,8]. The platform’s integration with Rwanda’s existing health care infrastructure represented a significant commitment to leveraging technology for improved health outcomes, underscored by the Government of Rwanda’s initial 10-year partnership agreement with the platform [8].

The scale of Babyl’s implementation was remarkable, reaching 450 out of 510 primary health facilities across Rwanda, representing over 80% of the primary health care market coverage before it halted in September 2023 as the system is being redesigned. By August 2020, the platform had enrolled 2 million patients, with approximately half having actively used the service to seek care. The service primarily catered to Community-Based Health Insurance (CBHI) members, who comprised over 80% of Babyl’s client base, alongside Rwandaise d’Assurance Maladie members and private clients. Rwandaise d’Assurance Maladie is the public medical insurance scheme that primarily covers employees in the public sector and also offers coverage to private sector employees and pensioners. It is coordinated by the Rwanda Social Security Board [9].

Despite the promising scale of implementation, the platform’s halt in September 2023 for system redesign highlighted critical gaps in understanding the complex factors that influence the adoption, utilization, and sustainability of large-scale digital health interventions. While previous studies, including Dalberg’s initial impact assessment, examined effects on access, equity, patient satisfaction, and cost-effectiveness compared with traditional care [7,10-12]. These evaluations did not provide insights into the deeper implementation challenges and user experiences that ultimately determine the success or failure of such platforms.

Notably, this study distinguishes itself from prior evaluations by examining the lived experiences and perspectives of diverse stakeholder groups, including active users, lapsed users, nonusers, health care providers, and Babyl agents, to understand the multifaceted barriers that contributed to the platform’s discontinuation. Unlike previous quantitative assessments focused primarily on utilization metrics and clinical outcomes, this qualitative investigation explores the contextual, social, and systemic factors that shaped user experiences and implementation outcomes. This study contributes to the literature through comprehensive stakeholder inclusion, implementation-focused analysis, and multi-level examination of factors influencing digital health platform adoption and sustainability.

The complexity of implementing digital health solutions extends beyond technical capabilities to encompass individual, community, and health system factors that can either facilitate or hinder adoption. Health care providers must adapt to new diagnostic approaches without physical examination capabilities, potentially altering clinical decision-making processes and provider-patient interactions [13,14]. Patients may face challenges in communicating illness concerns and treatment preferences through digital platforms, while also navigating new technologies that require different levels of digital literacy and access to reliable communication infrastructure.

Understanding these multifaceted implementation experiences becomes crucial as countries across sub-Saharan Africa and other low- and middle-income settings continue to explore and invest in digital health solutions [15,16]. The WHO Digital Health Guidelines emphasize the importance of understanding contextual factors and user experiences in digital health implementation [17]. The COVID-19 pandemic accelerated the global adoption of telemedicine and digital care models, making it imperative to learn from large-scale implementations like Babyl to inform future digital health initiatives [18].

This study uniquely addresses critical knowledge gaps by providing the first comprehensive, multi-stakeholder qualitative evaluation of Rwanda’s Babyl telemedicine platform, explicitly contrasting perspectives across patients (active users, lapsed users, registered nonusers, and eligible nonregistrants), health care providers, HC administrators, and Babyl agents. Unlike previous evaluations focused primarily on utilization metrics or single stakeholder groups, this study systematically examines the complex interplay between individual, community, health system, and technological factors shaping implementation outcomes, while explicitly linking demographic characteristics, including age, education, geographic location, and digital literacy, to adoption patterns and user experiences. These insights extend beyond Rwanda’s context to inform digital health implementation strategies across sub-Saharan Africa and other LMICs pursuing universal health coverage through technological innovations.

## Methods

### Study Design and Setting

This qualitative, cross-sectional study was conducted across Rwanda’s health care system to explore key drivers that supported or hindered the adoption and scale-up of Babyl’s digital health services in Rwanda. The study used focus group discussions (FGDs) and key informant interviews (KIIs) to capture perspectives from multiple stakeholder groups involved in or affected by the digital health platform implementation. The combination of FGDs and KIIs was strategically chosen to capture both collective community perspectives and in-depth individual experiences. FGDs enabled exploration of shared experiences, social norms, and community-level barriers through group interactions, while KIIs provided detailed accounts from health care providers and program implementers who could speak to operational challenges and system-level implementation issues. This methodological triangulation strengthened the validity and comprehensiveness of findings by allowing cross-verification of themes across different data sources and participant perspectives.

### Sampling Strategy and Participant Selection

A purposive, maximum variation sampling strategy was used to ensure representation of diverse experiences and perspectives across different contexts. The study used a multi-stage sampling approach. First, HCs were purposively selected based on three criteria: (1) Babyl service utilization rates (high, medium, and low), (2) geographic distribution across Rwanda’s provinces, and (3) presence or absence of Babyl agents at the facility level. This stratification ensured representation of diverse implementation contexts and user experiences.

Twelve HCs were selected across 10 districts, distributed as follows: two from the City of Kigali (urban), four from the Eastern Province (mixed urban-rural), three from the Southern Province (predominantly rural), two from the Western Province (rural), and one from the Northern Province (rural). This geographic distribution captured variations in infrastructure, digital connectivity, and socioeconomic contexts that might influence digital health service adoption.

Within each selected HC catchment area, recruitment occurred through multiple channels. HC staff identified potential participants from patient registers and community health worker networks. Community meetings were held at centrally located venues to explain the study’s purpose and answer questions. Interested individuals were screened for eligibility using a structured screening form. Recruitment continued until data saturation was achieved and the target sample size was reached. For FGDs, four distinct user categories were identified:

1. Active users: CBHI members who had registered for and actively used Babyl services within the past 6 months.
2. Lapsed users: CBHI members who had registered but stopped using Babyl services.
3. Registered nonusers: CBHI members who registered for Babyl but never used the services.
4. Eligible nonregistrants: CBHI members aware of Babyl services but never registered.

Participants were eligible if they were 18 years or older, CBHI members in the selected catchment areas, and able to provide informed consent. For KII participants, eligibility criteria included being used at the selected HC or serving as a Babyl agent for at least 6 months, ensuring adequate experience with the platform implementation. For KIIs, three categories of key informants were purposively selected:

1. HC heads: Administrative leaders overseeing HC operations.
2. Health care providers: Nurses, clinical officers, and physicians who interacted with Babyl-referred patients.
3. Babyl agents: Community-based facilitators who assisted users with platform registration and navigation.

The final sample size of 192 participants (160 in FGDs and 32 in KIIs) was determined based on several criteria: (1) Data saturation confirmed when no new themes emerged from the final 3 FGDs and 5 KIIs; (2) Representation of all four user categories across diverse geographic settings; (3) Multi-site coverage, including stakeholders from all 12 HCs; (4) Methodological guidelines suggesting 15‐20 FGDs for thematic saturation in multi-site studies; and (5) Distribution of 4‐5 FGDs per user category. The sample size exceeded the minimum recommendations.

### Data Collection and Procedures

Data collection was conducted between October 2022 and January 2023 by a team of 6 trained research assistants with prior qualitative research experience. All data collectors underwent a 3-day training covering qualitative interviewing techniques, research ethics, study objectives, and the semi-structured interview guides. Mock interviews were conducted to standardize data collection approaches across team members.

The research team comprised public health researchers with backgrounds in health systems strengthening, digital health, and implementation science. The principal investigator is a PhD holder with 8 years of experience in health policy research. The study coordinator is a female MPH graduate with expertise in qualitative methods. Research assistants included 4 females and 2 males, all with bachelor’s degrees in public health. No team members had prior professional relationships with Babyl or the participants.

Semi-structured interview guides were developed based on a literature review, consultation with digital health experts, and the research team’s expertise in health systems and implementation science. The interview guides were adapted from WHO’s framework for digital health implementation research [19] and incorporated constructs from the Consolidated Framework for Implementation Research [20]. The guides were pretested with 5 users and 2 health care providers from a nonstudy HC. Based on feedback, questions were refined for clarity, cultural appropriateness, and to reduce technical jargon.

The guides covered key domains including: (1) awareness and perceptions of digital health services, (2) facilitators and barriers to adoption, (3) user experiences with service delivery processes, (4) satisfaction with care quality, (5) perceived benefits and challenges, and (6) recommendations for improvement. Content validity was established through expert review by 3 implementation science researchers and 2 clinicians with telemedicine experience. Interview guides are provided as Multimedia Appendices 1-4.

Each FGD included 6‐10 participants of the same user category to promote open discussion and ensure homogeneity within groups. FGDs were conducted in Kinyarwanda (local language) at community meeting spaces near the HCs, lasted 60‐90 minutes, and were audio-recorded with participant consent. Two team members facilitated each FGD: one as moderator and one as note-taker to ensure comprehensive documentation of verbal and nonverbal data.

KIIs were conducted individually in private rooms at HCs or Babyl agent offices, lasted 45‐60 minutes, and were also audio-recorded. Interviews were scheduled at times convenient for participants to minimize disruption to their work responsibilities.

Field notes were taken during all interviews to capture contextual information, nonverbal cues, and preliminary observations. A reflexive journal was maintained by the research team lead to document potential biases, decision-making processes, and emerging insights throughout data collection.

In addition to qualitative data, demographic and contextual information were collected from all participants using structured questionnaires administered prior to FGDs and KIIs. For FGD participants, the demographic questionnaire captured age, gender, education level, geographic location (urban/rural), smartphone ownership status, digital literacy self-assessment, health insurance type, and Babyl service utilization history. For KII participants, the questionnaire captured professional role, years of professional experience, duration of Babyl involvement, health facility location, and service coverage characteristics.

### Data Analysis

All audio recordings were transcribed verbatim in Kinyarwanda within 48 hours of data collection by professional transcribers trained in qualitative transcription standards. Transcripts were then translated into English by bilingual team members. A back-translation process was used for 20% of transcripts to ensure translation accuracy and conceptual equivalence. Transcripts were anonymized, with all identifying information removed and replaced with participant codes.

Transcript quality was verified through a validation process where the lead researchers reviewed 30% of transcripts against audio recordings. Additionally, summary transcripts were shared with a subset of 15 participants for member checking to ensure accurate representation of their views.

Data were analyzed using iterative, inductive thematic analysis following Braun and Clarke’s 6-phase framework [18] : (1) familiarization with data, (2) generating initial codes, (3) searching for themes, (4) reviewing themes, (5) defining and naming themes, and (6) producing the report.

Two researchers independently coded the first 4 transcripts to develop an initial codebook. Codes were compared and discussed to achieve consensus, resulting in a refined codebook with clear code definitions. This codebook was then applied to all transcripts using NVivo (version 12; Lumivero) qualitative analysis software. Intercoder reliability was assessed on 20% of transcripts coded independently by 2 researchers, achieving a Cohen kappa of 0.82, indicating strong agreement.

The coding process began with open coding, where segments of text were assigned descriptive codes. A total of 127 initial codes were identified during open coding. These codes were then grouped into broader categories through axial coding, examining relationships and patterns. Finally, selective coding identified overarching themes that captured the essence of participants’ experiences and perspectives.

The analysis was conducted iteratively, with constant comparison between emerging themes and raw data. Deviant cases were actively sought and analyzed to ensure a comprehensive understanding. Regular team meetings were held to discuss emerging themes, resolve discrepancies, and refine the thematic structure.

Thematic saturation was formally assessed by monitoring theme emergence across sequential data collection phases. Data collection occurred in 3 waves. When analysis of the final 3 FGDs and 5 KIIs revealed no new themes, data collection was concluded.

Data credibility was enhanced through (1) methodological triangulation using both FGDs and KIIs, (2) investigator triangulation with multiple researchers involved in coding and analysis, (3) participant validation through member checking, (4) peer debriefing sessions with external qualitative research experts, and (5) maintaining a detailed audit trail documenting all analytical decisions.

Quantitative data were analyzed using descriptive statistics and chi-square tests to examine associations between demographic characteristics and technology access patterns. Statistical significance was set at *P*<.05. These quantitative findings are presented alongside qualitative data to provide a comprehensive understanding of barriers.

### Ethical Considerations

Ethical approval for this study was obtained from the University of Rwanda, College of Medicine and Health Sciences Institutional Review Board (approval number 335/CMHS IRB/2022). Additional administrative clearance was granted by the Rwanda Ministry of Health and participating district health offices. The study was conducted in accordance with the ethical principles outlined in the Declaration of Helsinki.

Written informed consent was obtained from all participants after providing detailed information about the study purpose, procedures, risks, benefits, and their right to withdraw at any time without consequences. For participants with limited literacy, the consent form was administered orally in the presence of a witness, and verbal consent was audio-recorded.

FGD participants received 5000 Rwandan francs (approximately US $5) as compensation for transportation and time. KII participants (health care providers, administrators, and Babyl agents) were interviewed during working hours with supervisor approval and did not receive monetary compensation. Refreshments were provided during all FGDs and interviews.

Participant privacy and confidentiality were ensured by conducting interviews in private settings, anonymizing transcripts, and storing audio recordings and transcripts on password-protected computers accessible only to the research team. Data will be securely stored for 5 years before permanent deletion. No adverse events were reported during the study.

## Results

### Overview

The analysis of 20 FGDs and 32 KIIs revealed five major themes that emerged from combining client and health facility personnel responses: (1) knowledge and perceptions of digital health services, (2) enablers and barriers to using digital services, (3) experience and satisfaction using Babyl digital services, (4) benefits of using digital services, and (5) suggestions to improve uptake of digital services. The KIIs also provided opportunities to better understand Babyl’s operations related to agent recruitment processes, client facilitation experiences, and engagement with health facilities.

### Participant Characteristics

The demographic distribution of FGD and KII participants is summarized in Tables1  2, respectively.

**Table 1.** Demographic characteristics of focus group discussion participants (n=160).

Characteristic	Active users (n=40)	Lapsed users (n=40)	Registered nonusers (n=40)	Eligible nonregistrants (n=40)
Sex, n (%)				
Female	25 (62.5)	27 (67.5)	28 (70.0)	21 (52.5)
Male	15 (37.5)	13 (32.5)	12 (30.0)	19 (47.5)
Age group (years), n (%)				
18‐30	12 (30.0)	8 (20.0)	6 (15.0)	10 (25.0)
31‐50	26 (65.0)	28 (70.0)	30 (75.0)	28 (70.0)
>50	2 (5.0)	4 (10.0)	4 (10.0)	2 (5.0)
Age (years), mean (SD)	35.2 (8.4)	36.8 (9.1)	38.4 (8.7)	42.1 (10.2)
Education level, n (%)				
Primary or less	10 (25.0)	15 (37.5)	25 (62.5)	28 (70.0)
Secondary	24 (60.0)	20 (50.0)	13 (32.5)	10 (25.0)
Tertiary	6 (15.0)	5 (12.5)	2 (5.0)	2 (5.0)
Location, n (%)				
Urban	11 (27.5)	9 (22.5)	10 (25.0)	6 (15.0)
Rural	29 (72.5)	31 (77.5)	30 (75.0)	34 (85.0)
Phone ownership, n (%)				
Own phone	38 (95.0)	36 (90.0)	33 (82.5)	27 (67.5)
Shared/no phone	2 (5.0)	4 (10.0)	7 (17.5)	13 (32.5)
Insurance type, n (%)				
CBHI^a^	35 (87.5)	32 (80.0)	33 (82.5)	34 (85.0)
Other/None	5 (12.5)	8 (20.0)	7 (17.5)	6 (15.0)

a CBHI: Community-Based Health Insurance.

**Table 2.** Characteristics of key informant interview participants (n=32).

Characteristic	Health center heads (n=6)	Health care providers (n=18)	Babyl agents (n=8)
Sex, n (%)			
Female	2 (33.3)	10 (55.6)	5 (62.5)
Male	4 (66.7)	8 (44.4)	3 (37.5)
Age group (years), n (%)			
25‐35	1 (16.7)	6 (33.3)	4 (50.0)
36‐45	3 (50.0)	9 (50.0)	3 (37.5)
>45	2 (33.3)	3 (16.7)	1 (12.5)
Age (years), mean (SD)	42.5 (7.2)	38.4 (6.8)	34.2 (5.9)
Professional role, n (%)			
Nurses	—^a^	12 (66.7)	—
Lab technicians	—	4 (22.2)	—
Pharmacists	—	2 (11.1)	—
Facility-based	6 (100)	—	8 (100)
Years of experience, n (%)			
<2	0 (0)	2 (11.1)	3 (37.5)
2‐5	1 (16.7)	8 (44.4)	5 (62.5)
6‐10	3 (50.0)	6 (33.3)	0 (0)
>10	2 (33.3)	2 (11.1)	0 (0)
Years of experience, mean (SD)	10.2 (4.8)	6.3 (4.2)	2.8 (1.5)
Health center type, n (%)			
Urban	2 (33.3)	7 (38.9)	3 (37.5)
Rural	4 (66.7)	11 (61.1)	5 (62.5)
Babyl experience (years), n (%)			
<1	1 (16.7)	4 (22.2)	1 (12.5)
1‐2	2 (33.3)	8 (44.4)	3 (37.5)
>2	3 (50.0)	6 (33.3)	4 (50.0)
Babyl experience (years), mean (SD)	2.4 (1.1)	1.8 (0.9)	2.1 (0.8)

a Not applicable.

A total of 192 participants were enrolled in this study, comprising 160 individuals in 20 FGDs and 32 key informants in individual interviews (KIIs). Among FGD participants, 98 (61.3%) were female, and 62 (38.7%) were male. The majority (112/160, 70.0%) were aged 31‐50 years, with 36 (22.5%) participants aged 18‐30 years, and 12 (7.5%) participants older than 50 years.

Demographic characteristics significantly influenced digital health adoption patterns. Twenty-eight younger participants (18‐30 y) demonstrated greater digital literacy and smartphone ownership (76.5%) compared with 4 participants older than 50 years (34.8%), facilitating easier platform adoption. Among the 12 participants older than 50 years, 11 (91.7%) participants reported difficulty navigating the digital platform. Female participants more frequently emphasized privacy as a key benefit, particularly for reproductive health consultations. Male participants more commonly highlighted time efficiency and workplace convenience. Rural participants (92/160, 57.5% of FGDs) faced compounded barriers: lower smartphone ownership (34.8% vs 76.5% in urban areas), poorer network connectivity (78.3% reported “poor” or “very poor” connection vs 23.5% urban), and limited Babyl agent presence (available in only 2 of 7 rural HCs vs all 5 urban centers).

Among active users (n=40), most were CBHI members (35/40, 87.5%), aged 25‐45 years, predominantly female (25/40, 62.5%), rural (29/40, 72.5%), and phone owners (38/40, 95%), with higher digital literacy and positive digital health experiences. Lapsed users (n=40) had similar demographic profiles but reported technology challenges and service dissatisfaction. Registered nonusers (n=40) were mainly female (28/40, 70%), older (mean age of 38 y), with lower digital literacy (25/40, 62.5% had only primary education), and largely rural (30/40, 75%). Eligible nonregistrants (n=40) were the oldest group (mean age of 42 y) with the lowest digital literacy and phone ownership (27/40, 67.5%), often relying on shared devices.

Table 2 shows that key informants had substantial professional experience and adequate exposure to Babyl services to provide informed perspectives. HC heads had the longest professional experience (mean 10.2 y), providing institutional memory and leadership perspective on digital health integration. Health care providers, predominantly nurses (12/18, 66.7%), had moderate experience (mean 6.3 y) and represented frontline staff managing the interface between digital and traditional services. Babyl agents were younger (mean age 34.2 y), had shorter professional tenure (mean 2.8 y), but substantial Babyl-specific experience (mean 2.1 y), positioning them to understand both technical platform issues and community-level barriers. The predominance of rural HCs (61.1%‐66.7% across groups) ensured representation of contexts where implementation challenges were most pronounced. Variation in Babyl experience duration (1‐2+ years across groups) captured perspectives from both early implementation and more mature operational phases.

Table 3 presents key findings organized by stakeholder group, revealing both convergent and divergent perspectives across patients/users, health care providers, HC heads, and Babyl agents. While all stakeholders recognized Babyl’s potential to improve health care access, critical disconnects emerged: patients emphasized front-end barriers, including registration complexity and agent availability; health care providers focused on back-end challenges including workflow integration and lack of access to consultation records; HC heads highlighted coordination difficulties and unclear protocols, and Babyl agents identified gaps in training, equipment, and support systems. These divergent priorities reflect each group’s position within the implementation ecosystem and underscore the importance of multi-stakeholder engagement in digital health design and implementation.

**Table 3.** Key findings by stakeholder group.

Theme	Patients/Users (n=160)	Health care providers (n=12)	Health center heads (n=6)	Babyl agents (n=14)
Knowledge and Perceptions	Mixed awareness levels; valued convenience but had an incomplete understanding of coverage and eligibility; trust concerns about remote diagnosis quality	Skepticism about diagnostic accuracy without physical examination; concerns about consultation quality and clinical decision-making limitations	Recognized potential for expanded access but concerned about facility burden, resource allocation, and unclear role in digital health integration	Strong belief in service value and community health impact; frustration when unable to assist users due to system limitations or lack of support
Enablers	Convenience and time savings; privacy for sensitive health issues; cost savings from avoided travel; access to qualified doctors; medication prescription and dispensing	Potential for reduced outpatient department congestion; access to physician consultation for complex cases; opportunity to refer patients requiring specialist input	Expanded health care access for the catchment population; potential to reduce referrals to district hospitals; alignment with national health coverage goals	Direct patient interaction and community engagement; satisfaction from helping users access health care; visible community health impact when services worked well
Barriers	Digital literacy gaps and limited smartphone access; agent absence at health centers; registration process complexity; poor network connectivity (especially rural); service coverage exclusions (chronic diseases, pediatrics)	No access to Babyl consultation records; medication stock-outs disrupting prescribed treatments; workflow disruptions from parallel service delivery; lack of feedback mechanisms to learn from consultations; diagnostic uncertainty without physical examination	Unclear integration protocols and coordination mechanisms; additional workload without corresponding resources; medication supply chain issues; ambiguous accountability when problems arose	Inadequate technical and patient education training; equipment failures and resource constraints; inconsistent presence at facilities; blamed for system issues beyond their control; limited escalation support
Experience and Satisfaction	High satisfaction when services functioned properly; frustration with technical issues, delays, and process complexity; pronounced age-related challenges (older adults required agent assistance 100%); lapsed users cited process friction exceeding perceived benefit	Frustration with incomplete patient information arriving at facilities; inability to provide continuity of care without consultation histories; diagnostic uncertainty affecting clinical confidence; felt Babyl operated as a parallel rather than an integrated system	Difficulty coordinating between Babyl and facility-based services; unclear accountability and escalation pathways when problems arose; concerned about sustainability and long-term resource implications	Satisfaction when helping users successfully; demotivation from facing blame for system failures; limited technical support for troubleshooting; felt undervalued despite frontline role
Recommendations	Universal agent availability at all health centers; simplified registration process; expanded service coverage (chronic diseases, pediatrics); improved rural network infrastructure; clearer information about eligibility and services	Full health management information system integration; access to consultation histories and prescribed treatments; regular coordination meetings with Babyl; clear referral and feedback protocols; assurance of medication availability	Clear operational guidelines and integration protocols; regular stakeholder coordination meetings; dedicated resources for digital health integration; sustainable financing clarity and commitment	Enhanced training (technical troubleshooting + patient education); reliable equipment and backup systems; consistent facility presence and defined roles; clear escalation procedures; recognition and support for frontline work

### Theme 1: Knowledge and Perceptions of Digital Health Services

#### Overview

Three common subthemes emerged: (1) sources of awareness about Babyl services, (2) perceptions of service quality and legitimacy, and (3) understanding of eligibility and coverage. These subthemes reveal how information dissemination and initial perceptions shaped enrollment decisions.

#### Sources of Awareness

Awareness of Babyl services came primarily through 3 channels. Health facility visits were the most common source, where patients learned about services during routine consultations or registrations. Radio advertisements were mentioned in 7 of 8 urban FGDs but only 3 of 8 rural FGDs, indicating geographic disparities in media reach. One participant stated,

*I heard about it from the radio. They said we can talk to doctors without going to the health center.*

[active user, FGD participant #3]

Word-of-mouth through community networks was particularly important in rural areas, where 6 of 8 FGDs cited neighbors or community health workers as information sources. Registered nonusers and eligible nonregistrants frequently mentioned incomplete or unclear information as a reason for nonengagement. As one participant explained*,*

*I heard about it, but I wasn’t sure if it was free with my insurance.*

[eligible nonregistrant, FGD participant #8]

#### Perceptions of Quality and Legitimacy

Perceptions of service quality varied substantially across user categories. Active users expressed confidence in the qualifications of Babyl doctors, emphasizing their ability to diagnose and prescribe appropriately. A typical comment was,

*They have doctors, real doctors who can prescribe medicine.*

[registered nonuser, FGD participant #2]

However, skepticism was common among nonregistrants and registered nonusers, who questioned whether remote consultations could match in-person care. This skepticism was more pronounced in rural areas (6 of 8 rural FGDs) compared with urban areas (3 of 8 urban FGDs) and among older participants (>50 y).

While 89% of CBHI members had heard of Babyl services, only 34% could accurately describe the service scope and eligibility. This awareness-understanding gap created adoption barriers across user groups. Active users learned through trial and error with agent assistance, while registered nonusers held persistent misunderstandings (eg, believing services required payment). Provider skepticism about remote diagnosis reinforced community doubts, creating a negative cycle where patients avoided services that providers did not endorse. This demonstrates that awareness campaigns alone are insufficient; comprehensive education addressing service scope, appropriate use cases, and complementarity with facility-based care is essential.

### Theme 2: Enablers and Barriers to Digital Service Utilization

#### Overview

Four subthemes emerged operating at different levels: (1) individual-level factors, including technology access and digital literacy, (2) community-level factors such as social norms and trust, (3) health system factors, including integration with existing services, and (4) technological infrastructure. Findings are organized by stakeholder perspectives to highlight divergent experiences.

#### Patient Perspectives on Enablers

Patients, particularly active users, identified several key enablers. Convenience and time savings were universally emphasized, with one participant noting,

*I don’t have to travel to the health center or wait in line.*

[active user, FGD participant #5]

Privacy emerged as a significant enabler, especially for female participants (mentioned in 68% of female vs 42% of male participant comments) and for sensitive health concerns. As one woman explained,

*I can get advice without anyone knowing my problem.*

[active user, FGD participant #9]

For working-age adults (31‐50 y), the ability to consult without missing work was critical.

*I don’t have to miss work to see a doctor.*

[active user, FGD participant #10]

Access to qualified doctors and prescribed medications was an additional enabler, particularly valued in rural areas where specialist access is limited.

#### Patient Perspectives on Barriers

Demographic and digital literacy data were collected using a structured questionnaire administered to all FGD participants (n=160). The questionnaire captured age, gender, education level, smartphone ownership, phone sharing patterns, and self-reported digital literacy. Quantitative data were analyzed using descriptive statistics and chi-square tests to examine associations between demographic characteristics and technology access patterns. Statistical significance was set at *P*<.05. These quantitative findings are presented alongside qualitative data to provide a comprehensive understanding of barriers.

Individual-level barriers were substantial and strongly correlated with demographic characteristics. Technology access challenges included a lack of smartphone ownership (affecting 65.2% of adults older than 50 y old vs 23.5% of 18‐30 y olds), reliance on shared devices (creating privacy concerns), and device limitations. One lapsed user described,

*My phone is very old and slow. The app takes too long to open.*

[lapsed user, FGD participant #2]

Smartphone ownership ranged from 76.5% (18‐30 y) to 34.8% (>50 y), *P*<.001. Rural participants older than 45 years had lower ownership (23.8%) than urban counterparts (71.4%, *P*<.001). Educational attainment predicted ownership: 85.7% secondary-educated versus 38.9% primary-educated owned smartphones (*P*<.001). Female participants were 2.3× more likely to share phones (62.5% vs 27.4% male, *P*<.001), creating privacy barriers for reproductive health discussions.

Digital literacy barriers were pronounced, with 91.7% of participants older than 50 years reporting difficulty using smartphone apps compared with 22.2% of 18‐30-year-olds. An eligible nonregistrant stated,

*I don’t know how to use these apps. I need someone to help me*.

[eligible nonregistrant, FGD participant #8]

Digital literacy showed a 4-fold age gradient: 91.7% of those older than 50 years needed continuous assistance versus 22.2% of 18‐30 years (*P*<.001). Educational patterns were equally pronounced: 89.7% primary-educated versus 23.4% secondary-educated required assistance (*P*<.001). All 8 participants, both older than 50 years and primary-educated, required help for every interaction, averaging 4.2 registration attempts.

Process complexity deterred many potential users, particularly the registration process, which required multiple steps, including phone verification, insurance validation, and profile setup.

*I tried to register but got confused with all the steps.*

[registered nonuser, FGD participant #6]

Trust concerns centered on data privacy, consultation quality without physical examination, and medication dispensing procedures. Service coverage limitations, particularly the exclusion of chronic diseases (diabetes, hypertension) and pediatric care, created significant barriers. Network connectivity issues disproportionately affected rural users, with 78.3% reporting poor or intermittent connections compared with 23.5% of urban users.

*The network doesn’t work well in my village.*

[eligible nonregistrant, FGD participant #3]

#### Health Care Provider Perspectives

Health care providers identified both enablers and barriers from their operational standpoint. Key enablers included reduced outpatient department (OPD) congestion for simple cases and access to physician expertise for complex cases. As one provider explained,

*For patients who only need simple consultations, this reduces our workload.*

[health care provider, KII]

However, providers raised concerns about diagnostic limitations, noting,

*Without physical examination, it’s difficult to make accurate diagnoses.*

[health center head, KII]

Workflow disruptions occurred when Babyl patients arrived at HCs without proper documentation or when consultation records were not accessible to facility staff.

*We don’t know what the doctor prescribed or why patients were referred.*

[health care provider, KII]

Medication stock-outs created frustration for both providers and patients.

*Sometimes the medication is out of stock when patients come to collect it.*

[health care provider, KII]

The lack of feedback mechanisms meant providers could not learn from Babyl consultations or understand clinical outcomes.

#### Babyl Agent Perspectives

Babyl agents identified community relationships and collaborative HC staff as key enablers, but faced significant operational barriers. Equipment inadequacy was a persistent challenge.

*Sometimes the equipment doesn’t work, and I have to use my own phone.*

[Babyl agent, KII]

Agents experienced blame for system failures beyond their control, affecting their motivation and community relationships.

*When the system fails, or verification takes long, patients blame me.*

[Babyl agent, KII]

Insufficient training on both technical troubleshooting and patient education limited their effectiveness. The absence of dedicated agents at many HCs (particularly rural facilities) was identified as a critical gap, with patients having no one to assist with registration or resolve issues.

Barriers compound multiplicatively for vulnerable populations. Older rural women with primary education faced simultaneously: limited smartphone ownership, agent absence, poor connectivity, registration complexity, and service coverage gaps. These disadvantages are concentrated among populations with the greatest potential benefit. While patients emphasized front-end barriers (registration, digital literacy, agent availability), providers focused on back-end challenges (workflow disruption, information access), indicating multilevel intervention needs. That 100% of participants older than 50 years required agent assistance, yet agents were absent from 41.7% of HCs, exemplifies how self-service assumptions created insurmountable barriers for vulnerable groups.

### Theme 3: Experience and Satisfaction Using Babyl Services

#### Overview

Three subthemes characterized user experiences: (1) registration and enrollment processes, (2) consultation quality and clinical interactions, and (3) factors influencing continued use or discontinuation. Experiences varied significantly by user category and demographic characteristics.

#### Patient Registration Experiences

Age was the strongest predictor of registration experience. Younger participants (18‐30 y) predominantly enrolled independently (89.5%), using smartphones and navigating the app without assistance.

*I was able to register easily on my own using the app.*

[active user, FGD participant #2]

In contrast, 100% of participants older than 50 years required agent assistance for registration.

*The agent helped me step by step to register and call.*

[active user, FGD participant #6]

This agent dependency created a critical vulnerability when agents were absent, older adults could not access services.

Registration success was age-stratified: 89.5% of 18‐30 years registered independently versus 0% of older than 50 years (100% required assistance). Educational attainment was equally predictive: 74.3% secondary-educated versus 15.6% primary-educated registered independently. Rural participants required 2.8 versus 1.3 registration attempts (urban, *P*<.001). Independent registrants were 3.7× more likely to initiate consultations (67.2% vs 18.1%), suggesting registration difficulty created psychological barriers to utilization.

#### Consultation Experiences and Discontinuation

Among lapsed users, the most common pattern was initial satisfaction with clinical outcomes but frustration with process complexity, leading to discontinuation.

*The medicine worked, but the process was too confusing to use again.*

[lapsed user, FGD participant #9]

Circumstantial discontinuation occurred when users’ health needs fell outside service coverage, particularly for chronic disease management.

*I tried it once for a cough, but now I just go to the health center.*

[lapsed user, FGD participant #2]

Registered nonusers typically completed enrollment but never initiated consultations due to persistent trust concerns or lack of immediate need.

#### Provider and Agent Experiences

Health care providers reported operational challenges, including the inability to access consultation records, medication stock-outs disrupting prescribed treatments, and a lack of clinical feedback limiting learning opportunities. Babyl agents experienced satisfaction when assisting successful users but felt blamed for system failures beyond their control, including verification delays and network connectivity problems.

User experiences revealed distinct trajectories: active users progressed through initial challenges (2‐3 attempts) to routine utilization; lapsed users reported satisfaction but discontinued due to process friction; registered nonusers completed enrollment but never initiated consultations. Age predicted experience quality through process navigation capability; older adults required sustained agent assistance. Without agent availability, consultation initiation was impossible regardless of need severity. Provider experiences paralleled patient frustrations: initial enthusiasm diminished when medication stock-outs and inability to access consultation records created operational challenges.

### Theme 4: Benefits of Digital Health Services

#### Overview

Four subthemes emerged regarding benefits: (1) access to specialized care and qualified providers, (2) time and cost savings, (3) health system efficiency improvements, and (4) innovation and learning opportunities. Benefits were emphasized differently by different stakeholder groups.

#### Patient-Identified Benefits

Patients most frequently emphasized access to doctors and specialists.

*I talked to a real doctor from Kigali who understood my problem.*

[active user, FGD participant #4]

This access benefit was particularly valued in rural areas where specialist consultations require traveling to district or referral hospitals. Time savings were mentioned more frequently by male participants (72%) and working-age adults than by female or older participants. Cost savings, including transport, lost work time, and informal payments, were emphasized more in rural FGDs (7 of 8) compared with urban FGDs (3 of 8).

*I saved money on transport and time away from my business.*

[active user, FGD participant #9]

Privacy and convenience benefits allowed participants to seek care for sensitive health issues and at convenient times.

Benefit valuations varied by demographics. Male participants emphasized time savings more than females (72.6% vs 48.0%), while females emphasized privacy (68.4% vs 41.9%). Age influenced perceptions: younger participants valued convenience, middle-aged participants emphasized time/cost, and older participants valued doctor access. Rural participants saved 2000‐3000 RWF (approximately US $1.90-2.86) versus urban 500‐1000 RWF (approximately US $0.48-0.95) per avoided visit, reflecting greater travel distances.

#### Provider and Agent-Identified Benefits

Health care providers valued the ability to refer complex cases to physicians.

*Patients can consult doctors from Kigali for complex cases.*

[health center head, KII]

Reduced OPD congestion for routine consultations allowed providers to focus on patients requiring physical examination.

*We can focus on patients who really need physical examination.*

[health care provider, KII]

However, providers noted that the lack of feedback limited their learning from consultations. Babyl agents identified community health impact and the satisfaction of facilitating access as key benefits, noting that successful users often became service advocates.

Stakeholder benefit perceptions diverged critically. Patients emphasized individual benefits (time savings, cost reduction, and privacy); providers emphasized system-level advantages (reduced congestion and specialist access). However, providers’ anticipated benefits remained unrealized due to implementation gaps. Babyl operated in parallel to facility services; patients still presented for medication collection. Inability to access consultation records created fragmented care. This disconnect created sustainability challenges: patients experienced sufficient benefit to continue utilization, but providers experienced more burdens than benefits, threatening institutional buy-in essential for scaling.

### Theme 5: Suggestions for Service Improvement

#### Overview

Four subthemes emerged regarding improvement recommendations: (1) community-level awareness and engagement strategies, (2) health facility-level infrastructure and staffing, (3) service delivery modifications, and (4) policy and sustainability considerations. Recommendations varied by stakeholder role and context.

#### Community-Level Recommendations

Expanded awareness campaigns using multiple channels (radio, community meetings, health facility outreach) were universally recommended.

*They should announce it at community meetings in villages.*

[eligible nonregistrant, FGD participant #1]

Participants suggested deploying community health worker mobilizers in cells and villages, particularly in rural areas. Clear, simplified information about eligibility, covered services, costs, and registration steps was emphasized across all user categories.

#### Health Facility-Level Recommendations

The most critical recommendation was ensuring dedicated Babyl agents at all HCs, especially rural facilities.

*There should be an agent at every health center to help people.*

[lapsed user, FGD participant #4]

Additional needs included adequate equipment (smartphones, tablets, backup power), improved network infrastructure, comprehensive agent training covering technical troubleshooting and patient education, and better integration of Babyl consultation records with health facility systems.

#### Service Delivery Recommendations

Service expansion to include pediatric consultations (for caregivers) was frequently requested. Participants suggested accommodating phone sharing through mechanisms like designated family members or community phone points. Partnerships with health posts for last-mile service delivery in remote areas were proposed. Chronic disease coverage was the most common service expansion request.

*It should cover chronic diseases like diabetes and hypertension.*

[registered nonuser, FGD participant #1]

Rural network infrastructure improvement was critical.

*The network infrastructure in rural areas needs improvement.*

[Babyl agent, KII]

Simplified registration processes, medication stock management improvements, and patient feedback mechanisms were additional operational recommendations.

#### Policy and Sustainability Considerations

Health care providers and HC heads emphasized sustainable financing mechanisms, clear governance and accountability structures, and formal integration into national health policies and strategies. These systemic recommendations reflected concerns about program continuity and scale-up.

### Demographic Patterns in Digital Health Adoption

Demographics functioned as intersecting determinants, creating compounded barriers. Older rural women with primary education faced simultaneously: smartphone nonownership (65.2%), digital illiteracy (91.7% needed assistance), poor connectivity (78.3% rural), agent absence (5 of 7 rural facilities), and service coverage gaps. Successful adopters were advantaged: 74.6% secondary-educated (vs 29.7% nonregistrants, *P*<.001), 87.5% urban, 95.0% smartphone owners, mean age 33.2 years (vs 42.1, *P*<.001). Each additional disadvantage multiplicatively reduced adoption: 87.5% utilization (zero disadvantages) versus 12.3% (3‐4 disadvantages). Digital health primarily reached already-advantaged populations, potentially exacerbating inequities.

Thematic synthesis reveals critical interdependencies among findings that explain implementation outcomes and point toward multilevel intervention requirements. Knowledge gaps identified in theme 1 directly amplified barrier impacts documented in theme 2: participants unaware that Babyl agents could provide registration assistance perceived enrollment as impossible and never attempted it, while informed participants with equivalent digital literacy limitations successfully navigated identical technical challenges with agent support. Service experiences captured in theme 3 created feedback loops affecting subsequent adoption: positive experiences generated word-of-mouth promotion that encouraged community members’ service uptake (“My neighbor used it successfully, so I tried it too,” FGD #7), while negative experiences, particularly older adults’ repeated inability to access services without agents, circulated through social networks actively deterring potential users from attempting enrollment. Perceived benefits in theme 4 remained theoretical rather than experienced for most participants because theme 2 barriers prevented service access; the value proposition was sound, but inaccessibility meant benefits could not be realized. Provider integration challenges documented in theme 2 directly undermined patient-perceived benefits highlighted in theme 4: medication unavailability at HCs negated Babyl consultations’ convenience advantages when patients still required facility visits, while lack of clinical follow-up information prevented the care continuity that would optimize health outcomes. Participants’ improvement recommendations in theme 5 implicitly recognized these interdependencies: universal agent deployment simultaneously addresses theme 1 knowledge gaps and theme 2 barrier navigation challenges; health management information system integration resolves theme 2 provider workflow challenges while enabling theme 3 positive experiences through care continuity; expanded service coverage addresses theme 2 coverage gaps while enhancing theme 4 benefit realization. The finding that no single intervention type dominated stakeholder recommendations participants proposed community-level awareness strategies, facility-level agent deployment and equipment improvements, service-level coverage expansion and process simplification, and policy-level financing and governance solutions—indicates that stakeholders intuitively understood that multilevel implementation challenges require correspondingly multilevel solutions. This systems perspective, emerging organically from participants’ lived experiences rather than being imposed by implementation science frameworks, suggests that user-centered digital health design must attend to complex interactions among individual capabilities, community resources, health system structures, technological infrastructure, and policy environments.

## Discussion

### Principal Findings

This qualitative study provides insights into the user experiences and implementation lessons from Babyl’s large-scale digital health platform in Rwanda, representing one of the most extensive telemedicine implementations in sub-Saharan Africa. Despite Babyl’s impressive reach, covering 450 of 510 primary health facilities and enrolling 2 million patients, the eventual closure in September 2023 underscores the critical importance of understanding implementation challenges beyond initial uptake metrics [7]. Importantly, the findings from this study are now informing Rwanda’s current redesign of its digital health strategy, providing evidence-based insights for developing more sustainable and effective digital health interventions. The findings reveal a complex interplay of individual, community, health system, and technological factors influencing digital health adoption, utilization, and sustainability, contributing valuable lessons for future initiatives in similar contexts and supporting Rwanda’s broader health system strengthening goals [21,22].

This study advances digital health implementation science through three distinct contributions to the literature. First, the comprehensive stakeholder inclusion approach sampled diverse groups, including active users, lapsed users (who discontinued services), registered nonusers (who never used despite registering), eligible nonregistrants (who were aware but never registered), health care providers, HC heads, and Babyl agents. Unlike previous evaluations that focused primarily on active users or clinical outcomes [7,10-12], this comprehensive sampling reveals the full spectrum of adoption patterns and identifies barriers across the complete user journey from awareness through sustained utilization. This methodological approach demonstrates that evaluating only successful adopters creates selection bias that obscures critical implementation barriers.

Second, the implementation-focused analytical framework examined why and how implementation succeeded or failed across different contexts, rather than solely measuring what outcomes were achieved. By examining facilitators, barriers, and contextual factors through an implementation science lens informed by WHO digital health guidelines [19] and the Consolidated Framework for Implementation Research [20], the study provides actionable insights for future digital health initiatives. This focus on implementation processes complements outcome-focused evaluations by revealing the mechanisms underlying utilization patterns.

Third, the multi-level analysis examined implementation challenges at individual levels (user characteristics, digital literacy), community levels (social norms, agent availability), health system levels (provider readiness, workflow integration), and policy levels (financing, governance structures). This holistic framework demonstrates that digital health implementation challenges cannot be addressed through single-level interventions. The finding that 100% of participants older than 50 years required agent assistance, yet 41.7% of HCs lacked agents, exemplifies how individual-level needs and system-level resource allocation interact to determine access outcomes. This multi-level perspective provides a comprehensive understanding of the complex, interacting factors that influenced Babyl’s trajectory and ultimate discontinuation.

The findings reveal profound equity considerations that must inform future digital health implementations. The demographic analysis demonstrates that digital health adoption is not merely a matter of individual choice but is fundamentally shaped by structural inequities in age, education, gender, and geography. Older adults faced substantial barriers, including limited smartphone ownership and digital literacy challenges. Educational attainment strongly predicted utilization patterns, with active users more likely to have secondary or higher education. Geographic disparities were pronounced, with rural participants experiencing lower smartphone ownership and substantially worse network connectivity. Gender differences emerged particularly in privacy concerns, with female participants emphasizing confidential consultations more frequently than male participants. These overlapping disadvantages create compounded barriers for the most vulnerable populations; older, less educated, rural, female participants face multiple simultaneous obstacles to digital health access. Future digital health interventions must proactively address these equity considerations through targeted strategies, including age-appropriate interfaces, low-literacy design, substantial rural infrastructure investments, and gender-sensitive implementation approaches.

Participants’ experiences highlight the critical importance of digital literacy support and trusted community channels for adoption. The finding that older participants consistently required agent assistance for registration, while younger participants more often enrolled independently, demonstrates that digital health platforms cannot rely on user self-service alone.

The absence of dedicated Babyl agents at many HCs, identified as the most critical implementation gap across all stakeholder groups, meant that older and less digitally literate potential users had no pathway to access services. Participants’ emphasis on word-of-mouth promotion and community health worker involvement reflects the importance of trusted local channels for health information dissemination [8]. Reports of users deleting verification codes due to cyberfraud concerns illustrate how broader digital security anxieties can undermine adoption, suggesting that digital health implementations require accompanying digital literacy and security education components [23].

Health care provider perspectives revealed critical health system integration gaps that limited clinical effectiveness and provider buy-in. Providers reported an inability to access Babyl consultation records, creating continuity of care challenges and workflow disruptions when patients arrived at facilities. The frequent medication stock-outs for prescribed treatments undermined patient confidence and created provider frustration. Lack of feedback mechanisms meant providers could not learn from Babyl consultations or understand clinical outcomes, limiting professional development opportunities. While providers valued reduced OPD congestion for simple cases and access to physician expertise for complex cases, concerns about diagnostic limitations without physical examination and parallel rather than integrated service delivery prevented full provider engagement. These findings demonstrate that technical platform functionality is insufficient; digital health implementations require genuine integration with clinical workflows, information systems, and pharmaceutical supply chains [5,17].

Digital health implementation in LMICs faces distinct challenges, including health care provider shortages, limited infrastructure access, inadequate education opportunities, greater disease burden, and insufficient data management capabilities compared with high-income countries [24,25]. Babyl’s experience can be contextualized within broader LMIC digital health implementations. India’s e-Sanjeevani platform delivered over 10 million teleconsultations through government-led integration within the national digital health strategy, while Kenya’s M-TIBA platform connected 4 million users with private-sector financing models integrated into existing health insurance schemes. Brazil’s Portal Telemedicina demonstrates infrastructure integration connecting remote primary care providers with specialists nationwide [24,25]. Unlike these government-integrated approaches, Babyl operated as a parallel system with limited health management information system integration and donor-dependent financing. Participants’ emphasis on national health system integration and expanded insurance coverage reflects awareness of structural requirements for digital health sustainability.

The findings highlight critical service design considerations for future implementations. The exclusion of chronic disease management (diabetes, hypertension) and pediatric consultations created significant coverage gaps, with many lapsed users discontinuing after initial acute illness treatment when ongoing health needs fell outside service scope. Participants’ recommendations for telephone sharing mechanisms and health post partnerships for remote areas reflect practical solutions to device access barriers. The registration process complexity, requiring multiple steps including phone verification, insurance validation, and profile setup, deterred potential users, particularly older adults and those with lower digital literacy. Successful registration required either independent technological capability or access to agent support; the absence of agents at many facilities meant the registration barrier was insurmountable for substantial population segments. These implementation details, often overlooked in digital health planning, proved critical to adoption and sustained utilization.

### Implementation Lessons and Policy Implications

The findings yield several implementation lessons for future digital health initiatives in similar contexts. First, sustainable financing mechanisms integrated within health system structures are essential. Babyl’s closure despite technical success and user satisfaction demonstrates that donor-dependent models face inherent sustainability challenges. Second, comprehensive health system integration from implementation outset is critical; parallel systems create workflow disruptions, continuity of care gaps, and provider resistance that undermine effectiveness regardless of platform technical capabilities. Third, differentiated implementation approaches are required for diverse population segments; one-size-fits-all strategies fail to address the distinct needs of older versus younger, rural versus urban, and high versus low digital literacy users. Fourth, adequate human resource allocation for user support is nonnegotiable. The absence of agents at HCs created insurmountable barriers for populations most in need of support. Finally, explicit governance frameworks clarifying digital health service accountability within existing health management structures are essential to prevent implementation confusion and ensure sustainability [2,3,12].

### Strengths and Limitations

This study has several strengths. The purposive sampling strategy across 4 distinct user categories (active users, lapsed users, registered nonusers, and eligible nonregistrants) enabled examination of the full adoption spectrum rather than only successful users, revealing critical barriers typically missed in implementation evaluations. Multi-stakeholder inclusion of patients, health care providers, HC heads, and Babyl agents provided triangulated perspectives on implementation facilitators and challenges from different vantage points within the health system. Geographic diversity across 6 districts with both urban and rural representation ensured findings captured varied contexts. Data collection timing during active implementation enabled exploration of real-time experiences rather than retrospective recall. The use of established qualitative methods with rigorous analysis procedures, including independent coding, regular team discussions, member checking, and audit trails, enhanced analytical validity [18].

Several limitations should be considered when interpreting findings. (1) The study relied on self-reported experiences and perceptions, which may be subject to recall bias and social desirability response patterns, particularly when discussing reasons for non-adoption or discontinuation. (2) Data collection occurred during Babyl’s active implementation period before closure was announced; participants’ perceptions may have differed had they known about impending service discontinuation. (3) Eligible nonregistrants who were unaware of Babyl services were not included, meaning perspectives from the least-reached population segments are absent. (4) The study did not directly assess clinical outcomes, cost-effectiveness, or health impact, limiting conclusions to user experiences and implementation processes rather than clinical or economic outcomes. (5) Although data saturation was documented through iterative data collection and analysis, the possibility remains that additional perspectives may exist within the broader population. (6) The specific context of Rwanda’s health system, insurance coverage structures, and telecommunications infrastructure may limit direct generalizability to other LMICs, though conceptual transferability to similar contexts is likely. (7) Language translation from Kinyarwanda to English may have introduced subtle meaning shifts despite back-translation validation procedures. (8) Selection bias may have occurred despite purposive sampling efforts, as participants willing to discuss digital health services may differ from those who declined participation. (9) The cross-sectional design captured experiences at a single time point; longitudinal follow-up would have enabled examination of evolving perceptions and utilization patterns over time.

### Conclusions

This qualitative study explored user experiences and implementation challenges of Babyl’s telemedicine platform in Rwanda, which operated from 2016 until its closure in September 2023. Our findings reveal that while participants recognized specific benefits of digital consultation, particularly convenience, reduced travel costs, and privacy, multiple interconnected barriers at individual, community, health system, and policy levels impeded widespread adoption and sustained utilization. Through analysis of experiences from active users, lapsed users, registered nonusers, and eligible nonregistrants, alongside perspectives from health care providers, HC heads, and Babyl agents, the study identified 5 major implementation themes that collectively explain the platform’s challenges.

Key insights from participants emphasize that successful digital health implementation extends beyond technological functionality to encompass critical human and structural elements. First, participants across all categories emphasized that digital literacy support and human assistance are nonnegotiable requirements, not optional enhancements. All participants (100%) older than 50 years required agent assistance, yet agent absence at HCs was identified as the single most critical implementation gap. Second, health care providers consistently reported that the platform operated as a parallel system rather than an integrated component of health service delivery, creating workflow disruptions, continuity of care gaps, and medication stock-out frustrations that undermined both provider satisfaction and patient confidence. Third, participants highlighted significant equity implications: older adults, individuals with lower educational attainment, rural residents, and those lacking smartphones faced compounded barriers that the service design failed to adequately address.

These findings carry practical implications for Rwanda’s current digital health redesign process and for similar initiatives in comparable contexts. Participants’ recommendations, validated across stakeholder groups, converge on five critical requirements: (1) genuine integration with existing health information systems and clinical workflows rather than parallel operation; (2) sustainable financing models embedded within national health system structures rather than donor-dependent arrangements; (3) universal availability of trained agents providing enrollment and troubleshooting support; (4) differentiated implementation strategies that accommodate varying levels of digital literacy, device access, and infrastructure; and (5) explicit governance frameworks clarifying roles, responsibilities, and accountability within health management structures. While this study focused specifically on Babyl’s experience in Rwanda, these implementation lessons may inform digital health initiatives in settings with similar health system structures, resource constraints, and digital infrastructure development stages.

## Supplementary material

10.2196/84832 Multimedia Appendix 1 Focus Group Discussion Guide for Babyl registered clients who never used digital services and nonregistered eligible CBHI (Community-Based Health Insurance) members who are aware of Babyl services.

10.2196/84832 Multimedia Appendix 2 Key Informant interview guide for health care providers.

10.2196/84832 Multimedia Appendix 3 Key Informant interview guide for Babyl agents.

10.2196/84832 Multimedia Appendix 4 Key Informant interview guide with health center staff.

## Abbreviations

CBHI: Community-Based Health Insurance
FGD: focus group discussion
HC: health center
KII: key informant interview
LMIC: low- and middle-income country
OPD: outpatient department
RAMA: Rwandaise d'Assurance Maladie
WHO: World Health Organization
