# Supplementary material for: Digital Primary Health in Rwanda: Qualitative Study of User Experiences and Implementation Lessons From Babyl’s Telemedicine Platform
Source: J Med Internet Res. 2026 Apr 1;28:e84832. doi: 10.2196/84832 (PMC13041620; doi:10.2196/84832)
Supplement: Multimedia Appendix 1 [file jmir-v28-e84832-s001.docx]

**EVALUATION OF INTEGRATED DIGITAL PRIMARY HEALTH CARE: THE CASE OF BABYL IN RWANDA**

**FORMATIVE EVALUATION TOOLS**

**Interview Guide for FGD of:**

- **Babyl registered clients who never used digital services**
- **Non-registered eligible CBHI members who are aware of Babyl services**

To begin the interview:

- ***First explain the consent form and get it signed.***
- ***Fill in the demographic information sheet (see attached)***

##### Introduction

Thank you again for accepting to be part of the study. To guide our interview today, I will ask a series of questions. As key issues arise, I may also ask follow-up questions to you. Your viewpoints are valuable, so I encourage you to speak up and share your thoughts. There is no need to come to consensus on any answer. You should try to answer and comment as accurately and truthfully as possible. I and the other focus group participants would appreciate it if you would refrain from discussing the comments of other group members outside the focus group. If there are any questions or discussions that you do not wish to answer or participate in, you do not have to do so; however please try to answer and be as involved as possible.

As I told you in the consent form, we have scheduled a maximum of 90 minutes for our discussion today and we would like to record this discussion because we don't want to miss any of your comments and want to make sure that our notes are accurate. Again, we will keep the recording private and no one except the research team will have access to the information documented during the discussion.

##### Ground rules/ Guidelines

- The most important rule is that only one person speaks at a time. There may be a temptation to jump in when someone is talking but please wait until they have finished.
- There are no right or wrong answers, only differing points of view.
- You do not have to speak in any particular order.
- When you do have something to say, please do so freely. There are many of you in the group and it is important that I obtain the views of each of you.
- You do not need to agree with others, but you must listen respectfully as others share their views.
- Rules for cellular phones: We ask that you put your phones in silent mode. If you must respond to a call, please step out of the room to do so and rejoin us as quickly as you can.
- Are there any questions before we begin? (*Address any questions the participant has)*
- Would it be okay for us to start recording? (If the participants agree, start recording the interview/discussion.)

##### Questions

###### Knowledge

- 1. Could you tell us what was your impression (or the community) the first time you/they heard about Babyl services? Probing questions:
     - What do you know about Babyl services?
     - How did you learn about Babyl services Where and when?

###### Enabling factors

1. What are the factors that motivated and enabled you/community to register / to enroll with Babyl? Probe questions:
   - Having a mobile phone
   - Availability of a Babyl agent at the health facility (Were you enrolled at a health care facility?)
   - Effect on travel time
   - Saving time, less queuing time at the facility
   - Consultation time, consultation privacy
   - Reduced cost associated with travel, and no co-payment
   - Experience from family members or friends who have used Babyl services
2. Are there some differences in the uptake and use of Babyl services between young and old people, men and women, people living in urban versus rural areas, across education levels? If yes, why?
3. Have you sought care at a conventional health care facility since registering with Babyl? Probe questions:
   - Why did you choose conventional care over Babyl?

###### Challenges/Barriers

1. What are the challenges/barriers that prevented you/other patients from enrolling and using Babyl services? Probing questions:
   - At individual level: familiarity / fear of using technology or digital healthcare services, lack of interest in using technology, feeling that care is impersonal, lack of trust, network availability, airtime (credit), electricity availability, phone ownership, gender?
   - Challenges/barriers at community level: rumors, cultural and religious beliefs, myths.
   - At Babyl service level: complex patient journey (enrolment, appointment, consultation, lab test, referral to health facilities and prescriptions) that ends up at the conventional care facility? Trouble accessing Babyl’s line, triage?

###### Experiences from family members, friends who have used Babyl services

You may have family members or friends, neighbors who have used Babyl’s services, and we would like to ask you the following questions:

1. What are the reasons that encourage Babyl clients to use and continue using its services?
2. What are the reasons that led Babyl clients to discontinue the use of Babyl’s services?

##### Suggestions for improvement

1. What can be done to increase the uptake and continuation of using Babyl digital health services in your communities? Probing questions
2. Suggestions at individual level
   - Suggestions at community level
   - Suggestions at health center level
   - Suggestions at Babyl project level
3. Would you like to discuss anything else related to Babyl digital services that we have not discussed?

##### Conclusion

- Thank you for participating. This has been a very successful discussion. Your opinions are valuable. We hope you have found the discussion interesting.
- If there is anything, you are unhappy with or wish to complain about, please contact the Principal Investigator or speak to me later.
- I would like to remind you that any comments and feedback are confidential and anything you share will help Babyl services in Rwanda.

Thank you!
