# Supplementary material for: Digital Primary Health in Rwanda: Qualitative Study of User Experiences and Implementation Lessons From Babyl’s Telemedicine Platform
Source: J Med Internet Res. 2026 Apr 1;28:e84832. doi: 10.2196/84832 (PMC13041620; doi:10.2196/84832)
Supplement: Multimedia Appendix 2 [file jmir-v28-e84832-s002.docx]

**EVALUATION OF INTEGRATED DIGITAL PRIMARY HEALTH CARE: THE CASE OF BABYL IN RWANDA**

**FORMATIVE EVALUATION TOOL**

##### Key Informant Interview Guide with Babyl health care providers

To begin the interview:

− First explain the consent form and get it signed.

#### INTRODUCTION

Thank you again for accepting to be part of the study. To guide our interview today, I will ask a series of questions. As key issues arise, I may also ask follow-up questions to you. Your viewpoints are valuable, so I encourage you to speak up and share your thoughts. There is no need to come to consensus on any answer. You should try to answer and comment as accurately and truthfully as possible.

Once again, thank you for taking the time to meet with me today. I want to talk about Babyl health services and the work you have been doing at this facility. Our team is conducting an evaluation to understand the factors that support or hindered the adoption and scale-up of Babyl digital health services in the country and more specifically in the area where you work.

As I told you in the consent form, we have scheduled 30 to 45 minutes for our discussion today and we would like to record this discussion because we don't want to miss any of your comments and want to make sure that our notes are accurate. Again, we will keep the recording private and no one except the research team will have access to the information documented during the discussion.

- Do you have any questions before we begin?
- Would it be okay for us to start recording? (If the participant is in agreement, start recording the interview/discussion).

#### QUESTIONS

###### Introduction

1. What is / are your qualification (s)? How long have you been working as a health care provider? How long have you been working at Babyl?
2. Do you work part or full time for Babyl? If part time, how much time per week do you dedicate to this activity?
3. Do you also work in a conventional healthcare facility? If so, which one? How often in a week/month? If no, have you worked at a conventional health care facility in the past?
4. What does your shift schedule look like at Babyl? How long is a typical shift? Are there days/times you typically work? How many patients do you attend to per week? How long is each consultation? How does this compare to a typical shift worked by you or your peers in a conventional healthcare facility?
5. What are your key responsibilities as Babyl health care provider? Can you tell us about your typical day at Babyl in details (activities that you are in charge of)?

###### Training received from Babyl

1. Were you trained for Babyl service provision? Could you describe the training you received-who organized and led it, for how long, what topics were covered during the training? Do you receive the in-service trainings organized by Babyl?
2. Are there any aspects of the training or supervision that you think should be improved in future training? If yes, explain.

###### Experience with Babyl services (offering services to Babyl clients)

1. Could you take us through the patient journey (enrolment, appointment, consultation, lab tests and prescriptions) when consulting with Babyl?

Probing questions:

- - Consultation: Do Babyl’s patients have to go through consultation again at the health center? What proportion do consult again at the heath center? For what reasons?
  - Are the USSD codes used for lab and drugs prescriptions well understood by patients and health facilities? Are there some patients who don’t receive? What do you do in such cases?
  - How often can someone use Babyl services? Are there any restrictions? For the same illness episode? For a different illness episode?

1. How would you compare your experience treating patients through Babyl to treating patients in-person? Probing questions:
   - What do you find easier or harder through Babyl?
   - Are there conditions that are easier (harder) to diagnose or treat through Babyl?
   - Are there any mistakes that you or other HCPs make more (less) often on Babyl than in-person?
   - Are there any other differences?
2. How easy is it to find patient information and follow up patients through the Babyl system?
3. What is your perception and clients’ perceptions on quality of care provided through digital interaction with patients?

###### Benefits of using Babyl services

1. What are the benefits of using Babyl services? Probing questions:

What is your opinion on: Saving time? Avoiding queuing at the health facility? Saving on transportation costs? Access to quality health services provided by healthcare providers (including doctors)?

###### Satisfaction with Babyl services

1. To what extent are Babyl patients satisfied or unsatisfied with Babyl services? Probing questions:

What are patients satisfied or unsatisfied with?

- - Babyl services in terms of ease of access and use of digital healthcare services (Babyl platform)?
  - Enrolment, appointment, consultation, lab test and prescriptions?
  - Quality of care and interaction with Babyl healthcare providers?
  - Anything else that we did not mention?

###### Factors facilitating or hindering the use of Babyl digital services

1. Based on your experience, what factors facilitate or support the use of Babyl services? Probing questions
   - At individual / patient level: Age? Education level? Gender? Phone ownership? Cheaper services?
   - At community level: Distance to health facility? Urban versus rural location?
   - At health facility level: Presence of a Babyl agent? Prevent queuing for consultation?
   - At Babyl level: Quality of services, interactions with providers, qualified and experienced staff?
2. What are the factors that prevent potential clients from using Babyl services? What about factors leading to discontinuation of Babyl services?

Probing questions:

- - At individual level: Age? education level? Gender? Use of technology digital healthcare services (Babyl system)? Network issues? Airtime issues? Electricity availability? Telephone issues.
  - At Babyl: Complex Patient journey (enrolment, appointment, consultation, lab test, referral to health facilities and prescriptions)? Impersonal interaction with the providers?
  - Challenges/barriers at community (rumors, cultural and religious beliefs, myths), health center (orientation when patients present USSD codes, relationship issues between Babyl agent and healthcare professionals) and Babyl service levels (accessing Babyl line call, triage, calling time, reception of medical prescription or lab tests)?
- Could the above-mentioned barriers/challenges be the reasons for the discontinuation of Babyl services? Please explain.
  - What are other reasons that could have triggered Babyl clients to discontinue using its services?

###### Suggestions for improvement

1. Are there any aspects of the training or supervision that you think should be improved in future training? If yes, explain.
2. Following your experience working with Babyl, what can be done to improve the Babyl operations and increase its uptake in this area?

Probing questions:

- - Suggestions at individual / user level
  - Suggestions at community level
  - Suggestions at health center level
  - Suggestions at Babyl project level

1. Would you like to discuss anything else related to Babyl digital services that we have not mentioned?

#### I. CONCLUSION

- Thank you for participating. This has been a very successful discussion. Your opinions are valuable. We hope you have found the discussion interesting.
- If there is anything, you are unhappy with or wish to complain about, please contact the Principal Investigator or speak to me later.
- I would like to remind you that any comments and feedback are confidential and anything you share will help Babyl services in Rwanda.

Thank you!!!!
