# Supplementary material for: Digital Primary Health in Rwanda: Qualitative Study of User Experiences and Implementation Lessons From Babyl’s Telemedicine Platform
Source: J Med Internet Res. 2026 Apr 1;28:e84832. doi: 10.2196/84832 (PMC13041620; doi:10.2196/84832)
Supplement: Multimedia Appendix 3 [file jmir-v28-e84832-s003.docx]

**EVALUATION OF INTEGRATED DIGITAL PRIMARY HEALTH CARE: THE CASE OF BABYL IN RWANDA**

**FORMATIVE EVALUATION TOOLS**

##### Key informant Interview Guide with Babyl agents

To begin the interview:

− First explain the consent form and get it signed.

#### INTRODUCTION

Thank you again for accepting to be part of the study. To guide our interview today, I will ask a series of questions. As key issues arise, I may also ask follow-up questions to you. Your viewpoints are valuable, so I encourage you to speak up and share your thoughts. There is no need to come to consensus on any answer. You should try to answer and comment as accurately and truthfully as possible.

Once again, thank you for taking the time to meet with me today. I want to talk about Babyl health services and the work you have been doing at this facility. Our team is conducting an evaluation to understand the factors that support or hindered the adoption and scale-up of Babyl digital health services in the country and more specifically in the area where you work.

As I told you in the consent form, we have scheduled 30 to 45 minutes for our discussion today and we would like to record this discussion because we don't want to miss any of your comments and want to make sure that our notes are accurate. Again, we will keep the recording private and no one except the research team will have access to the information documented during the discussion.

- Do you have any questions before we begin?
- Would it be okay for us to start recording? (If the participant is in agreement, start recording the interview/discussion)

#### QUESTIONS

###### Introduction

1. What is / are your qualification (s)? how long have you been working as a Babyl agent?
2. Can you share with us what you know about Babyl? Probing questions:
   - In your view, what are the key objectives of Babyl?
   - How is the Babyl project perceived among health care providers working at this health center?
3. What are your key responsibilities as a Babyl agent? Can you describe in detail your daily activities?
4. Are there any Babyl awareness activities that are conducted at this health center or in its catchment area? If yes, what are these awareness activities? Who conduct them? How often do they happen? Are you involved in these activities? If yes, how?
5. What are different materials and equipment received from Babyl project to read a pharmacy prescription or lab tests (e.g.: Computers, tablets, etc.…)

###### Experience facilitating / supporting Babyl services

1. How easy is it to enroll new Babyl users at the health care facilities? Probing questions:
   - How often do eligible patients decline to enroll in Babyl? Do they give reasons? If yes, what are these reasons? If no, what could be the reasons?
   - Are there particular types of people who are more or less likely to enroll when asked?
2. Would you share with us your experience facilitating Babyl services? Along every step of the process, highlight advantages and disadvantages of using Babyl services?

Probing questions:

- - Enrolment process: How do you compare patient enrolling/registering alone at home versus patient enrolling with Babyl agent support? In your opinion, what proportion of Babyl’s clients enroll through Babyl agents?
  - Booking process: How do you compare patients booking alone at home versus patients booking with Babyl agent support?
  - Consultation: Do Babyl’s patients have to go through consultation at the health center again? What proportion of Babyl patients do consult again at the heath center? For what reasons? Do Babyl’s registered clients continue to use conventional care services? What are the main reasons?
  - How do Babyl’s clients receive medical prescriptions and lab tests? What would you share with us about the USSD codes that are sent to Babyl patients for prescriptions and lab tests? Do you understand the received USSD codes? Are there some patients who don’t receive or don’t understand the USSD codes? How do Babyl agents support clients presenting USSD codes at the health facility?

###### Benefits of using Babyl services

1. What are the special benefits of using Babyl services? Probing questions:

What is your opinion on: Saving time? Avoiding queuing at the health facility? Saving on transportation costs? Access to quality health services provided by healthcare providers (including doctors)?

###### Satisfaction with Babyl services

1. To what extent are Babyl patients satisfied or unsatisfied with Babyl services? Probing questions:

What are patients satisfied or unsatisfied with?

- - Babyl services in terms of ease of access and use of digital healthcare services (Babyl platform)?
  - Enrolment, appointment, consultation, lab test and prescriptions?
  - Quality of care and interaction with Babyl healthcare providers?

###### Factors facilitating or hindering the use of Babyl digital services

1. Based on your experience, what factors facilitate or support the use of Babyl services? Probing questions
   - At individual / patient level: Age? Education level? Gender? Phone ownership? Cheaper services?
   - At community level: Distance to health facility? Urban versus rural location?
   - At health facility level: Presence of a Babyl agent? Prevent queuing for consultation?
   - At Babyl level: Quality of services, interactions with providers, qualified and experienced staff?

###### Challenges/Barriers in using Babyl services

1. What are the factors that prevent potential clients from using Babyl services? What about factors leading to discontinuation of Babyl services?

Probing questions:

- - At individual level: Age? education level? Gender? Use of technology digital healthcare services (Babyl system)? Network issues? Airtime issues? Electricity availability? Telephone issues.
  - Complex Patient journey (enrolment, appointment, consultation, lab test, referral to health facilities and prescriptions)? Impersonal interaction with the providers?
  - Challenges/barriers at community (rumors, cultural and religious beliefs, myths), health center (orientation when patients present USSD codes, relationship issues between Babyl agent and healthcare professionals) and Babyl service levels (accessing Babyl line call, triage, calling time, reception of medical prescription or lab tests)?
  - Could the above-mentioned barriers/challenges be the reasons for the discontinuation of Babyl services? Please explain.
    - What are other reasons that could have triggered Babyl clients to discontinue using its services?

###### Suggestions for improvement

1. What can be done to increase the uptake and continuation of using Babyl digital health services in your communities? Probing questions:
   - Suggestions at individual level
   - Suggestions at community level
   - Suggestions at health center level
   - Suggestions at Babyl project level
2. Would you like to discuss anything else related to Babyl digital services that we have not discussed?

###### CONCLUSION

- Thank you for participating. This has been a very successful discussion. Your opinions are valuable. We hope you have found the discussion interesting.
- If there is anything, you are unhappy with or wish to complain about, please contact the Principal Investigator or speak to me later.
- I would like to remind you that any comments and feedback are confidential and anything you share will help Babyl services in Rwanda.

THANK YOU!!!

**Interview Guide for FGD of:**

- **Babyl registered clients who never used digital services**
- **Non-registered eligible CBHI members who are aware of Babyl services**

To begin the interview:

- ***First explain the consent form and get it signed.***
- ***Fill in the demographic information sheet (see attached)***

##### Introduction

Thank you again for accepting to be part of the study. To guide our interview today, I will ask a series of questions. As key issues arise, I may also ask follow-up questions to you. Your viewpoints are valuable, so I encourage you to speak up and share your thoughts. There is no need to come to consensus on any answer. You should try to answer and comment as accurately and truthfully as possible. I and the other focus group participants would appreciate it if you would refrain from discussing the comments of other group members outside the focus group. If there are any questions or discussions that you do not wish to answer or participate in, you do not have to do so; however please try to answer and be as involved as possible.

As I told you in the consent form, we have scheduled a maximum of 90 minutes for our discussion today and we would like to record this discussion because we don't want to miss any of your comments and want to make sure that our notes are accurate. Again, we will keep the recording private and no one except the research team will have access to the information documented during the discussion.

##### Ground rules/ Guidelines

- The most important rule is that only one person speaks at a time. There may be a temptation to jump in when someone is talking but please wait until they have finished.
- There are no right or wrong answers, only differing points of view.
- You do not have to speak in any particular order.
- When you do have something to say, please do so freely. There are many of you in the group and it is important that I obtain the views of each of you.
- You do not need to agree with others, but you must listen respectfully as others share their views.
- Rules for cellular phones: We ask that you put your phones in silent mode. If you must respond to a call, please step out of the room to do so and rejoin us as quickly as you can.
- Are there any questions before we begin? (*Address any questions the participant has)*
- Would it be okay for us to start recording? (If the participants agree, start recording the interview/discussion.)

##### Questions

###### Knowledge

- 1. Could you tell us what was your impression (or the community) the first time you/they heard about Babyl services? Probing questions:
     - What do you know about Babyl services?
     - How did you learn about Babyl services Where and when?

###### Enabling factors

1. What are the factors that motivated and enabled you/community to register / to enroll with Babyl? Probe questions:
   - Having a mobile phone
   - Availability of a Babyl agent at the health facility (Were you enrolled at a health care facility?)
   - Effect on travel time
   - Saving time, less queuing time at the facility
   - Consultation time, consultation privacy
   - Reduced cost associated with travel, and no co-payment
   - Experience from family members or friends who have used Babyl services
2. Are there some differences in the uptake and use of Babyl services between young and old people, men and women, people living in urban versus rural areas, across education levels? If yes, why?
3. Have you sought care at a conventional health care facility since registering with Babyl? Probe questions:
   - Why did you choose conventional care over Babyl?

###### Challenges/Barriers

1. What are the challenges/barriers that prevented you/other patients from enrolling and using Babyl services? Probing questions:
   - At individual level: familiarity / fear of using technology or digital healthcare services, lack of interest in using technology, feeling that care is impersonal, lack of trust, network availability, airtime (credit), electricity availability, phone ownership, gender?
   - Challenges/barriers at community level: rumors, cultural and religious beliefs, myths.
   - At Babyl service level: complex patient journey (enrolment, appointment, consultation, lab test, referral to health facilities and prescriptions) that ends up at the conventional care facility? Trouble accessing Babyl’s line, triage?

###### Experiences from family members, friends who have used Babyl services

You may have family members or friends, neighbors who have used Babyl’s services, and we would like to ask you the following questions:

1. What are the reasons that encourage Babyl clients to use and continue using its services?
2. What are the reasons that led Babyl clients to discontinue the use of Babyl’s services?

##### Suggestions for improvement

1. What can be done to increase the uptake and continuation of using Babyl digital health services in your communities? Probing questions
2. Suggestions at individual level
   - Suggestions at community level
   - Suggestions at health center level
   - Suggestions at Babyl project level
3. Would you like to discuss anything else related to Babyl digital services that we have not discussed?

##### Conclusion

- Thank you for participating. This has been a very successful discussion. Your opinions are valuable. We hope you have found the discussion interesting.
- If there is anything, you are unhappy with or wish to complain about, please contact the Principal Investigator or speak to me later.
- I would like to remind you that any comments and feedback are confidential and anything you share will help Babyl services in Rwanda.

Thank you!

##### Key Informant Interview Guide with Health Center Staff (who has interacted significantly with Babyl’s referred patients, includes heads of HF, nurses, lab technicians and pharmacists)

To begin the interview:

− First explain the consent form and get it signed.

#### INTRODUCTION

Thank you again for accepting to be part of the study. To guide our interview today, I will ask a series of questions. As key issues arise, I may also ask follow-up questions to you. Your viewpoints are valuable, so I encourage you to speak up and share your thoughts. There is no need to come to consensus on any answer. You should try to answer and comment as accurately and truthfully as possible.

Once again, thank you for taking the time to meet with me today. I want to talk about Babyl health services and the work you have been doing at this facility. Our team is conducting an evaluation to understand the factors that support or hindered the adoption and scale-up of Babyl digital health services in the country and more specifically in the area where you work.

As I told you in the consent form, we have scheduled 30 to 45 minutes for our discussion today and we would like to record this discussion because we don't want to miss any of your comments and want to make sure that our notes are accurate. Again, we will keep the recording private and no one except the research team will have access to the information documented during the discussion.

Do you have any questions before we begin?

Would it be okay for us to start recording? (If the participant agrees, start recording the interview/discussion)

#### QUESTIONS

###### Introduction

1. Could you start by telling me a little about yourself? What is / are your qualification (s)? What is your position? How long have you been working at this health facility?
2. Can you share with us what you know about Babyl? Probing questions:
   - In your view, what are the key objectives of Babyl?
   - How is the Babyl project perceived among health care providers working at this health center?
   - Have you ever considered working as a Babyl provider? Why or why not? (If applicable)
3. What are your key responsibilities and activities as health care provider (nurse, lab technician / pharmacist)? How does this differ from your responsibilities and activities with regards to patients from Babyl? What are the main challenges faced while serving Babyl’s clients?
4. Are there any Babyl awareness activities that are conducted at this health center or in its catchment area? What are these awareness activities? How often do they happen? Are you involved in these activities? If yes, how?
5. Given your exposure to Babyl, what can you share about Babyl services delivery and referral of patients to your health center? Probing questions:
   - How do you compare health services offered through conventional care and Babyl care?
   - Consultation: Do Babyl’ s patients have to go through consultation again at the health center? What proportion do consult again at the heath center? Do they consult again the same day or within the same episode? For what reasons?
   - Are you able, at your facility to understand the received USSD codes for lab and drugs prescriptions? Are there some patients who don’t receive? Have you ever faced any issues with these codes?

###### Training and materials received from Babyl

1. What are the types of training received from Babyl to support clients treated through digital interaction and presenting at this health facility for lab testing and / or a pharmacy prescription?

Probing questions:

- - From what I understand you received an orientation/training for Babyl service provision? Could you describe the orientation/training for me?
  - What are different materials and equipment received from Babyl project to read a pharmacy prescription or lab tests (e.g.: Computers, tablets, etc.…)

1. Are there any aspects of the training or supervision that you think should be improved in future training? If yes, explain.

###### Experience with Babyl services (offering services to Babyl clients)

1. Imagine that you were a Babyl provider, offering services over the phone. How do you imagine your experience would differ through Babyl than treating patients in-person?

Probing questions:

- - Are there things you imagine would be easier (harder) through phone?
  - Are there conditions that would be easier (harder) to diagnose or treat through phone? Give some examples.
  - Are there any mistakes that you or other HCPs may make more (less) often on phone than in-person? What are they?
  - Are there any other differences that you can imagine?

1. Do your processes (e.g., administrative processes, administering and processing tests, filling prescriptions, reports, claims management, etc.) differ between Babyl and conventional health facility patients?

Probing questions:

- - At what stages in the process can you distinguish Babyl from conventional patients?
  - Are there any different protocols in place for Babyl patients?

1. What is your perception and /or perception of clients on quality of care provided through digital interaction with patients?

###### Benefits of using Babyl services

1. What are the special benefits of using Babyl services? Probing questions:

What is your opinion on: Saving time? Avoiding queuing at the health facility? Saving on transportation/ service costs? Access to quality health services provided by healthcare providers (including doctors)?

###### Satisfaction with Babyl services

1. To what extent are Babyl patients satisfied or unsatisfied with Babyl services? Probing questions:

What are patients satisfied or unsatisfied with?

- - Babyl services in terms of ease of access and use of digital healthcare services (Babyl platform)?
  - Enrolment, appointment, consultation, lab test and prescriptions?
  - Quality of care and interaction with Babyl healthcare providers?
  - Anything else that we did not mention?

###### Factors facilitating or hindering the use of Babyl digital services

1. Based on your experience, what factors facilitate or support the use of Babyl services? Probing questions
   - At individual / patient level: Age? Education level? Gender? Phone ownership? Cheaper services?
   - At community level: Distance to health facility? Urban versus rural location?
   - At health facility level: Presence of a Babyl agent? Prevent queuing for consultation?
   - At Babyl level: Quality of services, interactions with providers, qualified and experienced staff?

###### Suggestions for improvement

1. Following your experience working with Babyl, what can be done to improve the Babyl operations and increase its uptake in this area?

Probing questions:

- - Suggestions at individual / user level
  - Suggestions at community level
  - Suggestions at health center level
  - Suggestions at Babyl project level

1. Would you like to discuss anything else related to Babyl digital services that we have not mentioned?

###### CONCLUSION

- Thank you for participating. This has been a very successful discussion. Your opinions are valuable. We hope you have found the discussion interesting.
- If there is anything, you are unhappy with or wish to complain about, please contact the Principal Investigator or speak to me later.
- I would like to remind you that any comments and feedback are confidential and anything you share will help Babyl services in Rwanda.

THANK YOU!!!

##### Key Informant Interview Guide with Babyl health care providers

To begin the interview:

− First explain the consent form and get it signed.

#### INTRODUCTION

Thank you again for accepting to be part of the study. To guide our interview today, I will ask a series of questions. As key issues arise, I may also ask follow-up questions to you. Your viewpoints are valuable, so I encourage you to speak up and share your thoughts. There is no need to come to consensus on any answer. You should try to answer and comment as accurately and truthfully as possible.

Once again, thank you for taking the time to meet with me today. I want to talk about Babyl health services and the work you have been doing at this facility. Our team is conducting an evaluation to understand the factors that support or hindered the adoption and scale-up of Babyl digital health services in the country and more specifically in the area where you work.

As I told you in the consent form, we have scheduled 30 to 45 minutes for our discussion today and we would like to record this discussion because we don't want to miss any of your comments and want to make sure that our notes are accurate. Again, we will keep the recording private and no one except the research team will have access to the information documented during the discussion.

- Do you have any questions before we begin?
- Would it be okay for us to start recording? (If the participant is in agreement, start recording the interview/discussion).

#### QUESTIONS

###### Introduction

1. What is / are your qualification (s)? How long have you been working as a health care provider? How long have you been working at Babyl?
2. Do you work part or full time for Babyl? If part time, how much time per week do you dedicate to this activity?
3. Do you also work in a conventional healthcare facility? If so, which one? How often in a week/month? If no, have you worked at a conventional health care facility in the past?
4. What does your shift schedule look like at Babyl? How long is a typical shift? Are there days/times you typically work? How many patients do you attend to per week? How long is each consultation? How does this compare to a typical shift worked by you or your peers in a conventional healthcare facility?
5. What are your key responsibilities as Babyl health care provider? Can you tell us about your typical day at Babyl in details (activities that you are in charge of)?

###### Training received from Babyl

1. Were you trained for Babyl service provision? Could you describe the training you received-who organized and led it, for how long, what topics were covered during the training? Do you receive the in-service trainings organized by Babyl?
2. Are there any aspects of the training or supervision that you think should be improved in future training? If yes, explain.

###### Experience with Babyl services (offering services to Babyl clients)

1. Could you take us through the patient journey (enrolment, appointment, consultation, lab tests and prescriptions) when consulting with Babyl?

Probing questions:

- - Consultation: Do Babyl’s patients have to go through consultation again at the health center? What proportion do consult again at the heath center? For what reasons?
  - Are the USSD codes used for lab and drugs prescriptions well understood by patients and health facilities? Are there some patients who don’t receive? What do you do in such cases?
  - How often can someone use Babyl services? Are there any restrictions? For the same illness episode? For a different illness episode?

1. How would you compare your experience treating patients through Babyl to treating patients in-person? Probing questions:
   - What do you find easier or harder through Babyl?
   - Are there conditions that are easier (harder) to diagnose or treat through Babyl?
   - Are there any mistakes that you or other HCPs make more (less) often on Babyl than in-person?
   - Are there any other differences?
2. How easy is it to find patient information and follow up patients through the Babyl system?
3. What is your perception and clients’ perceptions on quality of care provided through digital interaction with patients?

###### Benefits of using Babyl services

1. What are the benefits of using Babyl services? Probing questions:

What is your opinion on: Saving time? Avoiding queuing at the health facility? Saving on transportation costs? Access to quality health services provided by healthcare providers (including doctors)?

###### Satisfaction with Babyl services

1. To what extent are Babyl patients satisfied or unsatisfied with Babyl services? Probing questions:

What are patients satisfied or unsatisfied with?

- - Babyl services in terms of ease of access and use of digital healthcare services (Babyl platform)?
  - Enrolment, appointment, consultation, lab test and prescriptions?
  - Quality of care and interaction with Babyl healthcare providers?
  - Anything else that we did not mention?

###### Factors facilitating or hindering the use of Babyl digital services

1. Based on your experience, what factors facilitate or support the use of Babyl services? Probing questions
   - At individual / patient level: Age? Education level? Gender? Phone ownership? Cheaper services?
   - At community level: Distance to health facility? Urban versus rural location?
   - At health facility level: Presence of a Babyl agent? Prevent queuing for consultation?
   - At Babyl level: Quality of services, interactions with providers, qualified and experienced staff?
2. What are the factors that prevent potential clients from using Babyl services? What about factors leading to discontinuation of Babyl services?

Probing questions:

- - At individual level: Age? education level? Gender? Use of technology digital healthcare services (Babyl system)? Network issues? Airtime issues? Electricity availability? Telephone issues.
  - At Babyl: Complex Patient journey (enrolment, appointment, consultation, lab test, referral to health facilities and prescriptions)? Impersonal interaction with the providers?
  - Challenges/barriers at community (rumors, cultural and religious beliefs, myths), health center (orientation when patients present USSD codes, relationship issues between Babyl agent and healthcare professionals) and Babyl service levels (accessing Babyl line call, triage, calling time, reception of medical prescription or lab tests)?
- Could the above-mentioned barriers/challenges be the reasons for the discontinuation of Babyl services? Please explain.
  - What are other reasons that could have triggered Babyl clients to discontinue using its services?

###### Suggestions for improvement

1. Are there any aspects of the training or supervision that you think should be improved in future training? If yes, explain.
2. Following your experience working with Babyl, what can be done to improve the Babyl operations and increase its uptake in this area?

Probing questions:

- - Suggestions at individual / user level
  - Suggestions at community level
  - Suggestions at health center level
  - Suggestions at Babyl project level

1. Would you like to discuss anything else related to Babyl digital services that we have not mentioned?

#### I. CONCLUSION

- Thank you for participating. This has been a very successful discussion. Your opinions are valuable. We hope you have found the discussion interesting.
- If there is anything, you are unhappy with or wish to complain about, please contact the Principal Investigator or speak to me later.
- I would like to remind you that any comments and feedback are confidential and anything you share will help Babyl services in Rwanda.

Thank you!!!!

##### Key Informant Interview Guide with Babyl health care providers

To begin the interview:

- - First explain the consent form and get it signed.

#### J. INTRODUCTION

Thank you again for accepting to be part of the study. To guide our interview today, I will ask a series of questions. As key issues arise, I may also ask follow-up questions to you. Your viewpoints are valuable, so I encourage you to speak up and share your thoughts. There is no need to come to consensus on any answer. You should try to answer and comment as accurately and truthfully as possible.

Once again, thank you for taking the time to meet with me today. I want to talk about Babyl health services and the work you have been doing at this facility. Our team is conducting an evaluation to understand the factors that support or hindered the adoption and scale-up of Babyl digital health services in the country and more specifically in the area where you work.

As I told you in the consent form, we have scheduled 30 to 45 minutes for our discussion today and we would like to record this discussion because we don't want to miss any of your comments and want to make sure that our notes are accurate. Again, we will keep the recording private and no one except the research team will have access to the information documented during the discussion.

- Do you have any questions before we begin?
- Would it be okay for us to start recording? (If the participant is in agreement, start recording the interview/discussion).

#### K. QUESTIONS

###### Introduction

1. What is / are your qualification (s)? How long have you been working as a health care provider? How long have you been working at Babyl?
2. Do you work part or full time for Babyl? If part time, how much time per week do you dedicate to this activity?
3. Do you also work in a conventional healthcare facility? If so, which one? How often in a week/month? If no, have you worked at a conventional health care facility in the past?
4. What does your shift schedule look like at Babyl? How long is a typical shift? Are there days/times you typically work? How many patients do you attend to per week? How long is each consultation? How does this compare to a typical shift worked by you or your peers in a conventional healthcare facility?
5. What are your key responsibilities as Babyl health care provider? Can you tell us about your typical day at Babyl in details (activities that you are in charge of)?

###### Training received from Babyl

1. Were you trained for Babyl service provision? Could you describe the training you received-who organized and led it, for how long, what topics were covered during the training? Do you receive the in-service trainings organized by Babyl?
2. Are there any aspects of the training or supervision that you think should be improved in future training? If yes, explain.

###### Experience with Babyl services (offering services to Babyl clients)

1. Could you take us through the patient journey (enrolment, appointment, consultation, lab tests and prescriptions) when consulting with Babyl?

Probing questions:

- - Consultation: Do Babyl’s patients have to go through consultation again at the health center? What proportion do consult again at the heath center? For what reasons?
  - Are the USSD codes used for lab and drugs prescriptions well understood by patients and health facilities? Are there some patients who don’t receive? What do you do in such cases?
  - How often can someone use Babyl services? Are there any restrictions? For the same illness episode? For a different illness episode?

1. How would you compare your experience treating patients through Babyl to treating patients in-person? Probing questions:
   - What do you find easier or harder through Babyl?
   - Are there conditions that are easier (harder) to diagnose or treat through Babyl?
   - Are there any mistakes that you or other HCPs make more (less) often on Babyl than in-person?
   - Are there any other differences?
2. How easy is it to find patient information and follow up patients through the Babyl system?
3. What is your perception and clients’ perceptions on quality of care provided through digital interaction with patients?

###### Benefits of using Babyl services

1. What are the benefits of using Babyl services? Probing questions:

What is your opinion on: Saving time? Avoiding queuing at the health facility? Saving on transportation costs? Access to quality health services provided by healthcare providers (including doctors)?

###### Satisfaction with Babyl services

1. To what extent are Babyl patients satisfied or unsatisfied with Babyl services? Probing questions:

What are patients satisfied or unsatisfied with?

- - Babyl services in terms of ease of access and use of digital healthcare services (Babyl platform)?
  - Enrolment, appointment, consultation, lab test and prescriptions?
  - Quality of care and interaction with Babyl healthcare providers?
  - Anything else that we did not mention?

###### Factors facilitating or hindering the use of Babyl digital services

1. Based on your experience, what factors facilitate or support the use of Babyl services? Probing questions
   - At individual / patient level: Age? Education level? Gender? Phone ownership? Cheaper services?
   - At community level: Distance to health facility? Urban versus rural location?
   - At health facility level: Presence of a Babyl agent? Prevent queuing for consultation?
   - At Babyl level: Quality of services, interactions with providers, qualified and experienced staff?
2. What are the factors that prevent potential clients from using Babyl services? What about factors leading to discontinuation of Babyl services?

Probing questions:

- - At individual level: Age? education level? Gender? Use of technology digital healthcare services (Babyl system)? Network issues? Airtime issues? Electricity availability? Telephone issues.
  - At Babyl: Complex Patient journey (enrolment, appointment, consultation, lab test, referral to health facilities and prescriptions)? Impersonal interaction with the providers?
  - Challenges/barriers at community (rumors, cultural and religious beliefs, myths), health center (orientation when patients present USSD codes, relationship issues between Babyl agent and healthcare professionals) and Babyl service levels (accessing Babyl line call, triage, calling time, reception of medical prescription or lab tests)?
- Could the above-mentioned barriers/challenges be the reasons for the discontinuation of Babyl services? Please explain.
  - What are other reasons that could have triggered Babyl clients to discontinue using its services?

###### Suggestions for improvement

1. Are there any aspects of the training or supervision that you think should be improved in future training? If yes, explain.
2. Following your experience working with Babyl, what can be done to improve the Babyl operations and increase its uptake in this area?

Probing questions:

- - Suggestions at individual / user level
  - Suggestions at community level
  - Suggestions at health center level
  - Suggestions at Babyl project level

1. Would you like to discuss anything else related to Babyl digital services that we have not mentioned?

#### L. CONCLUSION

- Thank you for participating. This has been a very successful discussion. Your opinions are valuable. We hope you have found the discussion interesting.
- If there is anything, you are unhappy with or wish to complain about, please contact the Principal Investigator or speak to me later.
- I would like to remind you that any comments and feedback are confidential and anything you share will help Babyl services in Rwanda.

Thank you!!!!
