# Supplementary material for: Digital Primary Health in Rwanda: Qualitative Study of User Experiences and Implementation Lessons From Babyl’s Telemedicine Platform
Source: J Med Internet Res. 2026 Apr 1;28:e84832. doi: 10.2196/84832 (PMC13041620; doi:10.2196/84832)
Supplement: Multimedia Appendix 4 [file jmir-v28-e84832-s004.docx]

**EVALUATION OF INTEGRATED DIGITAL PRIMARY HEALTH CARE: THE CASE OF BABYL IN RWANDA**

**FORMATIVE EVALUATION TOOL**

##### Key Informant Interview Guide with Health Center Staff (who has interacted significantly with Babyl’s referred patients, includes heads of HF, nurses, lab technicians and pharmacists)

To begin the interview:

− First explain the consent form and get it signed.

#### INTRODUCTION

Thank you again for accepting to be part of the study. To guide our interview today, I will ask a series of questions. As key issues arise, I may also ask follow-up questions to you. Your viewpoints are valuable, so I encourage you to speak up and share your thoughts. There is no need to come to consensus on any answer. You should try to answer and comment as accurately and truthfully as possible.

Once again, thank you for taking the time to meet with me today. I want to talk about Babyl health services and the work you have been doing at this facility. Our team is conducting an evaluation to understand the factors that support or hindered the adoption and scale-up of Babyl digital health services in the country and more specifically in the area where you work.

As I told you in the consent form, we have scheduled 30 to 45 minutes for our discussion today and we would like to record this discussion because we don't want to miss any of your comments and want to make sure that our notes are accurate. Again, we will keep the recording private and no one except the research team will have access to the information documented during the discussion.

Do you have any questions before we begin?

Would it be okay for us to start recording? (If the participant agrees, start recording the interview/discussion)

#### QUESTIONS

###### Introduction

1. Could you start by telling me a little about yourself? What is / are your qualification (s)? What is your position? How long have you been working at this health facility?
2. Can you share with us what you know about Babyl? Probing questions:
   - In your view, what are the key objectives of Babyl?
   - How is the Babyl project perceived among health care providers working at this health center?
   - Have you ever considered working as a Babyl provider? Why or why not? (If applicable)
3. What are your key responsibilities and activities as health care provider (nurse, lab technician / pharmacist)? How does this differ from your responsibilities and activities with regards to patients from Babyl? What are the main challenges faced while serving Babyl’s clients?
4. Are there any Babyl awareness activities that are conducted at this health center or in its catchment area? What are these awareness activities? How often do they happen? Are you involved in these activities? If yes, how?
5. Given your exposure to Babyl, what can you share about Babyl services delivery and referral of patients to your health center? Probing questions:
   - How do you compare health services offered through conventional care and Babyl care?
   - Consultation: Do Babyl’ s patients have to go through consultation again at the health center? What proportion do consult again at the heath center? Do they consult again the same day or within the same episode? For what reasons?
   - Are you able, at your facility to understand the received USSD codes for lab and drugs prescriptions? Are there some patients who don’t receive? Have you ever faced any issues with these codes?

###### Training and materials received from Babyl

1. What are the types of training received from Babyl to support clients treated through digital interaction and presenting at this health facility for lab testing and / or a pharmacy prescription?

Probing questions:

- - From what I understand you received an orientation/training for Babyl service provision? Could you describe the orientation/training for me?
  - What are different materials and equipment received from Babyl project to read a pharmacy prescription or lab tests (e.g.: Computers, tablets, etc.…)

1. Are there any aspects of the training or supervision that you think should be improved in future training? If yes, explain.

###### Experience with Babyl services (offering services to Babyl clients)

1. Imagine that you were a Babyl provider, offering services over the phone. How do you imagine your experience would differ through Babyl than treating patients in-person?

Probing questions:

- - Are there things you imagine would be easier (harder) through phone?
  - Are there conditions that would be easier (harder) to diagnose or treat through phone? Give some examples.
  - Are there any mistakes that you or other HCPs may make more (less) often on phone than in-person? What are they?
  - Are there any other differences that you can imagine?

1. Do your processes (e.g., administrative processes, administering and processing tests, filling prescriptions, reports, claims management, etc.) differ between Babyl and conventional health facility patients?

Probing questions:

- - At what stages in the process can you distinguish Babyl from conventional patients?
  - Are there any different protocols in place for Babyl patients?

1. What is your perception and /or perception of clients on quality of care provided through digital interaction with patients?

###### Benefits of using Babyl services

1. What are the special benefits of using Babyl services? Probing questions:

What is your opinion on: Saving time? Avoiding queuing at the health facility? Saving on transportation/ service costs? Access to quality health services provided by healthcare providers (including doctors)?

###### Satisfaction with Babyl services

1. To what extent are Babyl patients satisfied or unsatisfied with Babyl services? Probing questions:

What are patients satisfied or unsatisfied with?

- - Babyl services in terms of ease of access and use of digital healthcare services (Babyl platform)?
  - Enrolment, appointment, consultation, lab test and prescriptions?
  - Quality of care and interaction with Babyl healthcare providers?
  - Anything else that we did not mention?

###### Factors facilitating or hindering the use of Babyl digital services

1. Based on your experience, what factors facilitate or support the use of Babyl services? Probing questions
   - At individual / patient level: Age? Education level? Gender? Phone ownership? Cheaper services?
   - At community level: Distance to health facility? Urban versus rural location?
   - At health facility level: Presence of a Babyl agent? Prevent queuing for consultation?
   - At Babyl level: Quality of services, interactions with providers, qualified and experienced staff?

###### Suggestions for improvement

1. Following your experience working with Babyl, what can be done to improve the Babyl operations and increase its uptake in this area?

Probing questions:

- - Suggestions at individual / user level
  - Suggestions at community level
  - Suggestions at health center level
  - Suggestions at Babyl project level

1. Would you like to discuss anything else related to Babyl digital services that we have not mentioned?

###### CONCLUSION

- Thank you for participating. This has been a very successful discussion. Your opinions are valuable. We hope you have found the discussion interesting.
- If there is anything, you are unhappy with or wish to complain about, please contact the Principal Investigator or speak to me later.
- I would like to remind you that any comments and feedback are confidential and anything you share will help Babyl services in Rwanda.

THANK YOU!!!
